# Addition of transcranial direct current stimulation to quadriceps strengthening exercise in knee osteoarthritis: A pilot randomised controlled trial

**DOI:** 10.1371/journal.pone.0180328

**Published:** 2017-06-30

**Authors:** Wei-Ju Chang, Kim L. Bennell, Paul W. Hodges, Rana S. Hinman, Carolyn L. Young, Valentina Buscemi, Matthew B. Liston, Siobhan M. Schabrun

**Affiliations:** 1 School of Science and Health, Western Sydney University, Sydney, New South Wales, Sydney, Australia; 2 School of Health Sciences, Melbourne, The University of Melbourne, Melbourne, Victoria, Australia; 3 School of Health and Rehabilitation Sciences, The University of Queensland, Brisbane, Queensland, Australia; University of Bologna, ITALY

## Abstract

A randomised, assessor- and participant-blind, sham-controlled trial was conducted to assess the safety and feasibility of adding transcranial direct current stimulation (tDCS) to quadriceps strengthening exercise in knee osteoarthritis (OA), and provide data to inform a fully powered trial. Participants were randomised to receive active tDCS+exercise (AT+EX) or sham tDCS+exercise (ST+EX) twice weekly for 8 weeks whilst completing home exercises twice per week. Feasibility, safety, patient-perceived response, pain, function, pressure pain thresholds (PPTs) and conditioned pain modulation (CPM) were assessed before and after treatment. Fifty-seven people were screened for eligibility. Thirty (52%) entered randomisation and 25 (84%) completed the trial. One episode of headache in the AT+EX group was reported. Pain reduced in both groups following treatment (AT+EX: p<0.001, partial η^2^ = 0.55; ST+EX: p = 0.026, partial η^2^ = 0.18) but no between-group differences were observed (p = 0.18, partial η^2^ = 0.08). Function improved in the AT+EX (p = 0.01, partial η^2^ = 0.22), but not the ST+EX (p = 0.16, partial η^2^ = 0.08) group, between-group differences did not reach significance (p = 0.28, partial η^2^ = 0.052). AT+EX produced greater improvements in PPTs than ST+EX (p<0.05) (superolateral knee: partial η^2^ = 0.17; superior knee: partial η^2^ = 0.3; superomedial knee: partial η^2^ = 0.26). CPM only improved in the AT+EX group but no between-group difference was observed (p = 0.054, partial η^2^ = 0.158). This study provides the first feasibility and safety data for the addition of tDCS to quadriceps strengthening exercise in knee OA. Our data suggest AT+EX may improve pain, function and pain mechanisms beyond that of ST+EX, and provides support for progression to a fully powered randomised controlled trial.

## Introduction

Knee osteoarthritis (OA) is a prevalent and costly health problem with no known cure. Approximately 10% of people aged over 60 years experience significant pain, physical dysfunction and reduced quality of life as a result of knee OA, and this figure is rising rapidly [1]. The development of low cost, non-drug, non-surgical treatments to improve patient outcomes has been identified as a key priority area by people living with OA [2].

Strengthening exercise is the cornerstone of conservative management for knee OA and is recommended in all clinical guidelines internationally [3, 4]. Although exercise is effective in knee OA, meta-analyses indicate treatment benefits are at best, moderate, for pain and physical function, and small in quality of life [5]. Novel treatments that enhance the benefits of strengthening exercise through synergistic mechanistic effects are one avenue that might further improve exercise outcomes for people with knee OA.

Transcranial direct current stimulation (tDCS) is a non-invasive brain stimulation technique with the potential to enhance the effectiveness of exercise in knee OA. Weak direct currents are applied to the brain via scalp electrodes to increase (anodal stimulation) or decrease (cathodal stimulation) the excitability of neurons in the region below the electrode and in distant interconnected areas [6–8]. As increased cortical excitability in the primary motor cortex (M1) is associated with motor learning [9–12], anodal tDCS of M1 is thought to increase the brain’s responsiveness to the afferent input generated by subsequent treatments such as motor training and peripheral electrical stimulation, a phenomenon known as priming [13–15]. In addition, evidence from healthy individuals and groups with chronic pain suggests anodal tDCS applied to the primary motor cortex (M1) can reduce pain through modulation of pain processing in cortical and subcortical regions, facilitation of descending anti-nociceptive pathways, and induction of synaptic change, reminiscent of neuroplasticity, in underlying brain regions [16–19]. On this basis, applying anodal tDCS to M1 in addition to the established exercise therapy for knee OA has the potential to bolster the mechanistic and clinical effects of exercise through two mechanisms: i) ‘priming’ the brain to increase its responsiveness to the corticomotor benefits of exercise (e.g. increased cortical excitability, enhanced voluntary muscle activation, strength gains, improved muscle coordination and motor control) and/or; ii) additive and complementary effects on pain system function which has been argued as an outcome of exercise [20]. Thus, the combined application of tDCS and exercise may enhance mechanistic and clinical outcomes in knee OA. However, there has been no research investigating the effect of tDCS combined with exercise therapy in people with osteoarthritic pain.

Only one study has attempted to combine tDCS with exercise for treatment of chronic pain [21]. That study demonstrated greater decreases in pain intensity and anxiety, as well as a trend towards a greater reduction in depression, in individuals with fibromyalgia when tDCS was delivered during aerobic exercise than when tDCS or exercise were delivered alone. These data suggest that tDCS may bolster the effects of exercise in chronic pain.

This pilot randomised clinical trial aimed to: i) determine the safety, feasibility and patient-perceived response of adding tDCS to an exercise program for knee OA; and ii) provide data to inform a sample size calculation for a fully-powered trial based on trends of efficacy in pain, physical function and pain system function should these be observed.

## Participants and methods

This randomised, assessor- and participant-blinded, controlled trial was prospectively registered with the Australian and New Zealand Clinical Trials Registry: ACTRN12613001320741. Ethical approval was obtained from Western Sydney University’s Human Research Ethics Committee (H10184). All participants provided written informed consent.

### Participants

Individuals who met the criteria of the American College of Rheumatology (ACR) clinical classification for idiopathic knee OA [22] were recruited between September 2014 and August 2015 in Sydney, Australia. The post-intervention assessment of the trial was completed in November 2015. The ACR criteria include the presence of knee pain plus at least three of the following six items: age over 50 years, morning stiffness lasting less than 30 minutes, crepitus, bony tenderness, bony enlargement and no palpable warmth. A minimum pain score of 40 on a 100mm visual analogue scale (VAS) on walking in the past week was required. Exclusion criteria are detailed in the protocol paper (Supplementary S1 File) [23]. Participants were permitted to continue their usual medications for the duration of the trial. Medication type and dosage were recorded at the baseline assessment. Potential participants completed an on-line or telephone screening questionnaire to determine eligibility. Eligible individuals were contacted to confirm their willingness to participate and to arrange baseline assessment. A single investigator (W-JC), blinded to the group allocation of the participants, performed participant recruitment, screening, and testing.

### Procedures

Participants were randomly allocated to: 1) active tDCS plus exercise (AT+EX); or 2) sham tDCS plus exercise (ST+EX). The randomisation schedule was concealed in consecutively numbered, sealed opaque envelopes. An investigator not involved in recruitment and assessment prepared and provided the envelopes to the treating physiotherapists who revealed group allocation. Participants received 20 minutes of either active or sham tDCS immediately prior to 30 minutes of one-to-one supervised strengthening exercise, twice weekly for eight weeks (16 sessions). tDCS was applied before exercise therapy based on findings of greatest clinical benefit in individuals with stroke when tDCS is applied before, and not during or after, a second therapy [24]. Treatment duration was based on previous studies that reported that at least 12 supervised exercise sessions are required for optimum results in knee OA [25]. The knee with worst symptoms was assessed and treated if bilateral knee OA was present. Assessment and treatment were performed in the laboratory at Western Sydney University. Physiotherapists with more than five years experience were trained in tDCS and delivered both the tDCS (active and sham) and exercise therapies. All participants were instructed to complete home exercises twice per week.

#### tDCS

tDCS was delivered via two 35 cm^2^ surface sponge electrodes using a direct current stimulator (DC-STIMULATOR, neuroConn, Ilmenau, Germany) while participants sat quietly. The active electrode (anode) was placed over M1 contralateral to the side of the worst knee and the reference electrode (cathode) over the contralateral supraorbital region [26]. The primary motor cortex has emerged as one of the most effective and reliable sites for tDCS in the treatment of pain, producing improvements in pain analogous to those of FDA approved pharmaceuticals in other musculoskeletal pain conditions with considerably fewer side-effects [27]. Current intensity was ramped up (0 mA to 1 mA) and down (1 mA to 0 mA) over 10 seconds at the beginning and end of the stimulation period. The stimulation protocol was selected based on tDCS literature [28]. For sham stimulation, electrodes were placed in an identical position. Stimulation was turned on for 15 seconds, then off, to provide the initial itching sensation. Participants were informed that they may or may not perceive any sensation during stimulation [29]. The success of participant blinding was assessed at post-intervention assessment using a Yes/No response to a series of questions to determine whether treatment allocation was divulged to participants before completion of the trial [23].

#### Exercise therapy

Standardised quadriceps strengthening exercises (5 in total) known to be effective in knee OA were performed with ankle cuff weights or resistance bands where appropriate [5, 30]. Each exercise was performed in 3 sets of 10 repetitions with a 30s break between sets. The exercises are described in detail in the protocol paper [23]. The exercise program was progressed as defined in the protocol. The starting level and when to progress the exercise were determined for each individual by the treating physiotherapists based on participant feedback and the therapist’s clinical judgement. Cuff weights/resistance bands were given to participants to perform their supervised exercises (at the same dosage) at home. Home exercise diaries with instructions were provided for recording the number of sessions, the type and number of exercises performed and any adverse reactions. Diaries were collected at the post-intervention assessment.

### Measures

Baseline and post-intervention assessments were performed within one week of commencing or completing the 8-week treatment. *Feasibility* was measured as the: (i) number of treatment sessions attended by each participant, (ii) number of drop-outs in each treatment group, (iii) proportion of participants recruited from the total number screened, (iv) willingness of each participant to undergo therapy on an 11-point numerical rating scale (NRS) with ‘not at all willing’ at 0 and ‘very willing’ at 10 (baseline only), and (v) number of home exercise sessions completed. *Safety* was assessed as any adverse reaction reported upon verbal questioning by the treating physiotherapists at each session [31]. The description of any adverse reaction, its severity and duration and how the adverse reaction was managed were documented.

#### Pain, function and perceived effect of treatment

Pain and function were measured using: (i) a 100 mm VAS for pain on walking over the past week with terminal descriptors of ‘no pain’ (score 0 mm) and ‘worst pain imaginable’ (score 100 mm), (ii) the Western Ontario and McMaster Universities Osteoarthritis Index (WOMAC) pain (5 items, total score = 20) and physical function (17 items, total score = 68) subscales [32]. The reliability of the VAS in OA has been demonstrated [33]. The WOMAC is a disease-specific valid and reliable instrument for knee OA [34]. Participants’ perceived response to therapy was assessed at post-intervention assessment using a 7-point Likert scale ranging from “completely recovered” to “vastly worsened”.

#### Pain mechanisms

The protocol for each measure is described in detail in the protocol paper [23]. In brief, the following measures were made:

1. Pressure pain thresholds (PPTs) were measured at two remote sites: a) ipsilateral tibialis anterior (10 cm distal to the tibial tuberosity), b) ipsilateral extensor carpi radialis longus (10 cm distal to the lateral epicondyle of the humerus); and eight sites at the worst knee: c) inferomedial- 2 cm distal to the inferior medial edge of patella; d) inferolateral- 2 cm distal to the interior lateral edge of patella; e) lateral- 3 cm lateral the mid point of the lateral patellar border; f) superolateral- 2 cm proximal to the superior lateral edge of patella; g) superior- 2 cm proximal to the mid point of the superior patellar border; h) superomedial- 2 cm medial to the superior medial edge of patellar; i) medial- 3 cm medial to the mid point of the medial patellar border; and j) centre of the patella [35]. The average of three measurements at each site was used in the analysis. The reliability of PPT in OA knee has been demonstrated (ICC = 0.83 [0.72–0.90]) [36].
2. Heat pain thresholds (HPTs) were measured at the worst knee (medial knee joint line, patella and lateral knee joint line) and the ventral aspect of the forearm (10 cm distal from the elbow crest) on both sides. The average of three measurements at each site was used in the analysis. HPT measure has moderate reliability in OA knee (ICC = 0.77 [0.62–0.87]) [36].
3. CPM was examined as a change in pain perceived in one body region (test stimulation [TS], pressure pain threshold) as a result of pain induced in another body region (conditioned stimulation [CS], heat pain). Participants completed two trials in random order: i) TS at the worst knee and CS at the contralateral forearm; ii) TS at the contralateral forearm and CS at the ipsilateral forearm. The CPM paradigm has demonstrated good intrasession reliability (ICC > 0.75) [37].
4. Nociceptive flexor withdrawal reflex (NFR) was measured using surface stimulating electrodes applied at a retromalleolar location along the expected location of the sural nerve on the side of the worst knee. Recording electrodes were positioned over the belly of the biceps femoris muscle. The intensity needed to evoke a response in biceps femoris, indicating activation of the NFR, the latency of the onset of the NFR response, the EMG amplitude of the NFR response (quantified as the area of the root mean square amplitude between onset and offset of the response) and the subjective pain score on a NRS (0–10) experienced from the sural nerve stimulus were recorded. The NFR is a reliable experimental test (intersession CV_SEM_ = 16.9%, ICC = 0.82) [38].

### Data analysis

A CONSORT [39] diagram was used to describe the flow of the participants and to summarise the eligibility, recruitment and follow-up rates throughout the trial. *T*-tests were used for between-group comparisons of baseline characteristics. Data distribution was tested for skewness, kurtosis and normality (Shapiro-Wilk test) prior to conducting the *T*-tests. The analyses of pain, function and pain system function were performed according to intention-to-treat analysis. Missing data were not replaced. To confirm the appropriateness of the statistical analysis plan for a full randomised controlled trial, repeated Measures Analysis of Variance [40] were conducted to compare baseline and post-intervention scores for each outcome, in each group. An analysis of covariance (ANCOVA) was used to assess between-group changes in pain, function and pain mechanisms, where group allocation was the fixed factor and the corresponding baseline outcome values were covariates [41]. Prior to conducting the analysis of variance and covariance tests, the normality (Shapiro-Wilk test) and the homogeneity of variances were tested. Results are presented as means and standard deviations unless otherwise stated.

## Results

### Feasibility

**Fig 1:**
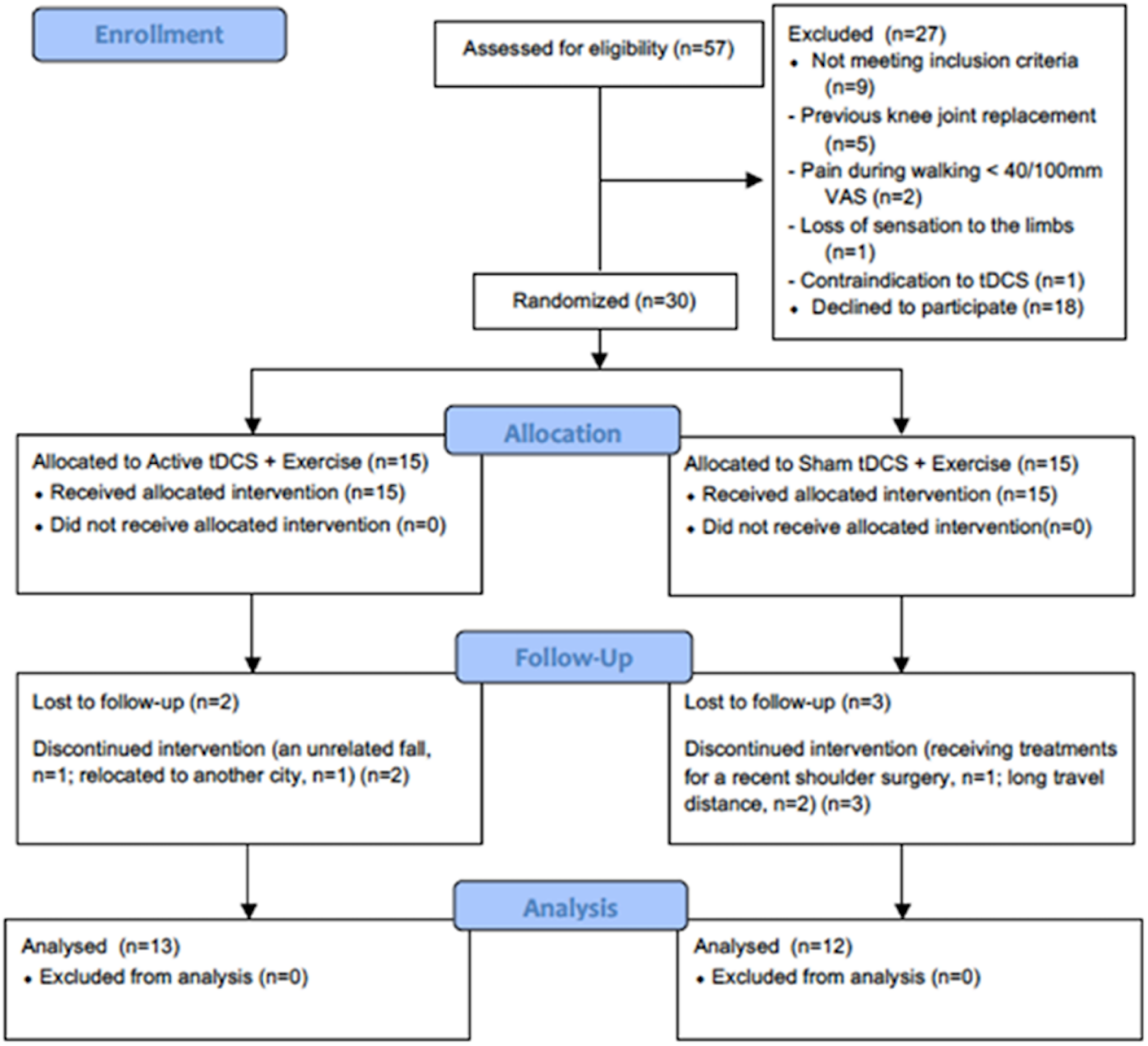
Consort diagram for flow of participants through the trial.

Fifty-seven people were screened for eligibility. Thirty-two (56%) met the inclusion criteria and attended baseline assessment. Two declined to participate after completing baseline assessment. Thirty screened participants (52%) were enrolled in the study and randomly allocated to a treatment group (Fig 1). Twenty-five enrolled participants (84%) (13 in the AT+EX group and 12 in the ST+EX group) completed the treatment and post-intervention assessment. The dropout rate was 16% (13% [n = 2] in the AT+EX group and 20% [n = 3] in the ST+EX group). In the AT+EX group, one participant withdrew after having an unrelated fall at home and the second relocated to another city. In the ST+EX group, one participant was unable to continue the study while simultaneously receiving physiotherapy after a rotator cuff repair surgery and two withdrew due to traveling distance required to attend treatments. The treatment attendance rate was 80% (14±1.7 sessions) in the AT+EX group and 78% (13.7±2.7 sessions) in the ST+EX group. The AT+EX group completed 14.7 (±2.3) home exercise sessions while the ST+EX group completed 11.3 (±5.2) sessions (out of 16). The demographic characteristics of all participants at baseline were similar between groups (Table 1). Blinding was successful; no participant reported that the type of tDCS stimulation was divulged before completing the post-intervention assessment. Eleven (73%) participants in the AT+EX group and seven (47%) in the ST+EX group correctly guessed their treatment group. The outcome assessor reported that the treatment allocation of participants was not divulged before the trial completion.

**Table 1:** Baseline characteristics of participants (mean and standard deviation).

	Active tDCS + Exercise (N = 15)	Sham tDCS + Exercise (N = 15)
**Age (year)**	59.8±9.1	64.1±11.1
**Gender (male/female)**	4/11	6/9
**Height (metre)**	1.6±0.08	1.6±0.11
**Weight (kg)**	89.0±13.3	84.5±16.4
**Body Mass Index (kg/metre**^**2**^**)**	31.3±3.5	30.5±9.1
**Duration of symptoms (years)**	7.2±5.3	9±7.3
**Previous knee arthroscopy**	4	6
**Bilateral OA knee pain**	12	10
**Side of worst knee pain (left/right)**	4/11	8/7
**Willingness to undergo treatment at baseline (out of 10)**	9.4±1.1	9.8±0.3
**Expected treatment effects**		
***Minimal improvement***	3(20%)	1(6%)
***Moderate improvement***	6(40%)	7(47%)
***Large improvement***	6(40%)	7(47%)
**Pain on walking (visual analog scale, 100 mm)**	59.8±15.2	56.4±19.7
**WOMAC *Total score***	55±16.0	48±10.7
***Pain***	11±3.9	9.9±3.2
***Physical function***	38.8±11.9	33.2±7.7

WOMAC = Western Ontario and McMaster Universities Osteoarthritis Index.

### Safety

One participant in the AT+EX group reported increased pain and swelling in her worst knee at week 6 of the treatment with no precipitating factors identified and was diagnosed with a first episode of gout (no previous history) by her general practitioner. She completed the trial after her symptoms settled. Two adverse reactions to tDCS were documented; one participant in the AT+EX group reported a single episode of headache after one treatment session and later withdrew from the study due to a fall at home. One participant in the ST+EX group reported a single incident of a painful sensation under the tDCS electrode when the current intensity was ramped up at the beginning of stimulation. tDCS was ceased immediately and the painful sensation resolved. The participant returned to complete the study after the incident and reported no further adverse reactions. No adverse reactions to, or concerns regarding the implementation of, the exercise program were identified.

### Perceived participant response to treatment

**Fig 2:**
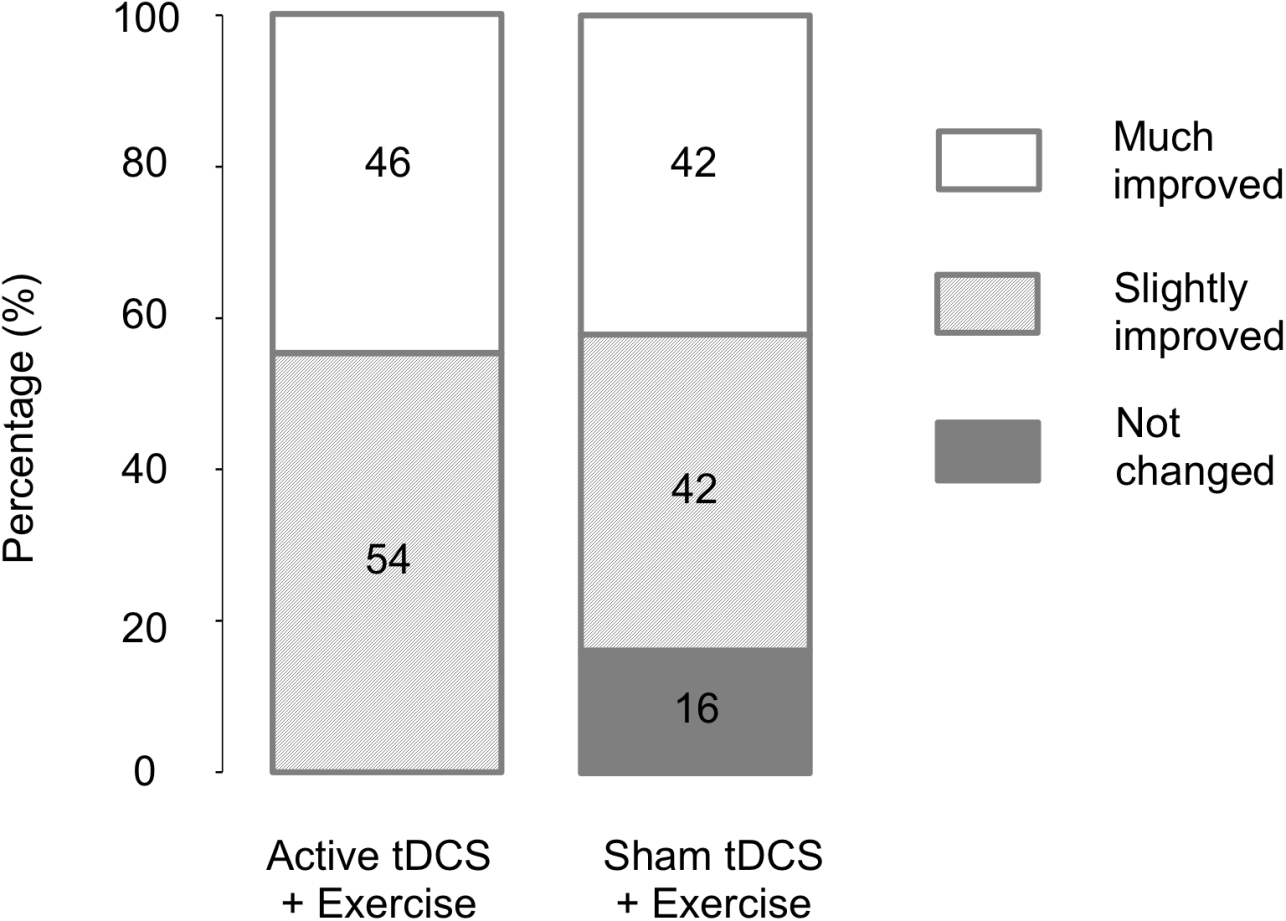
Percentage of participants reporting perceived improvement across categories from ‘not changed’ to ‘much improved’. Note: no participants reported that their condition worsened after either intervention.

All participants in the AT+EX group and 84% in the ST+EX group reported an improvement in their knee OA symptoms following treatment (Fig 2). No participant reported that knee symptoms worsened with either treatment.

### Pain and function

**Fig 3:**
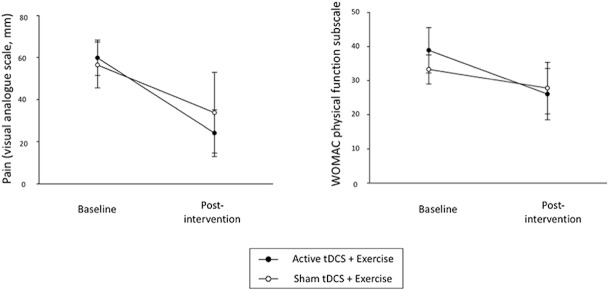
Pain and WOMAC physical function subscale (mean and 95% confidence interval) pre- and post-interventions. Active tDCS + exercise produced improvements in pain and function but sham tDCS + exercise only produced improvement in pain.

**Fig 4:**
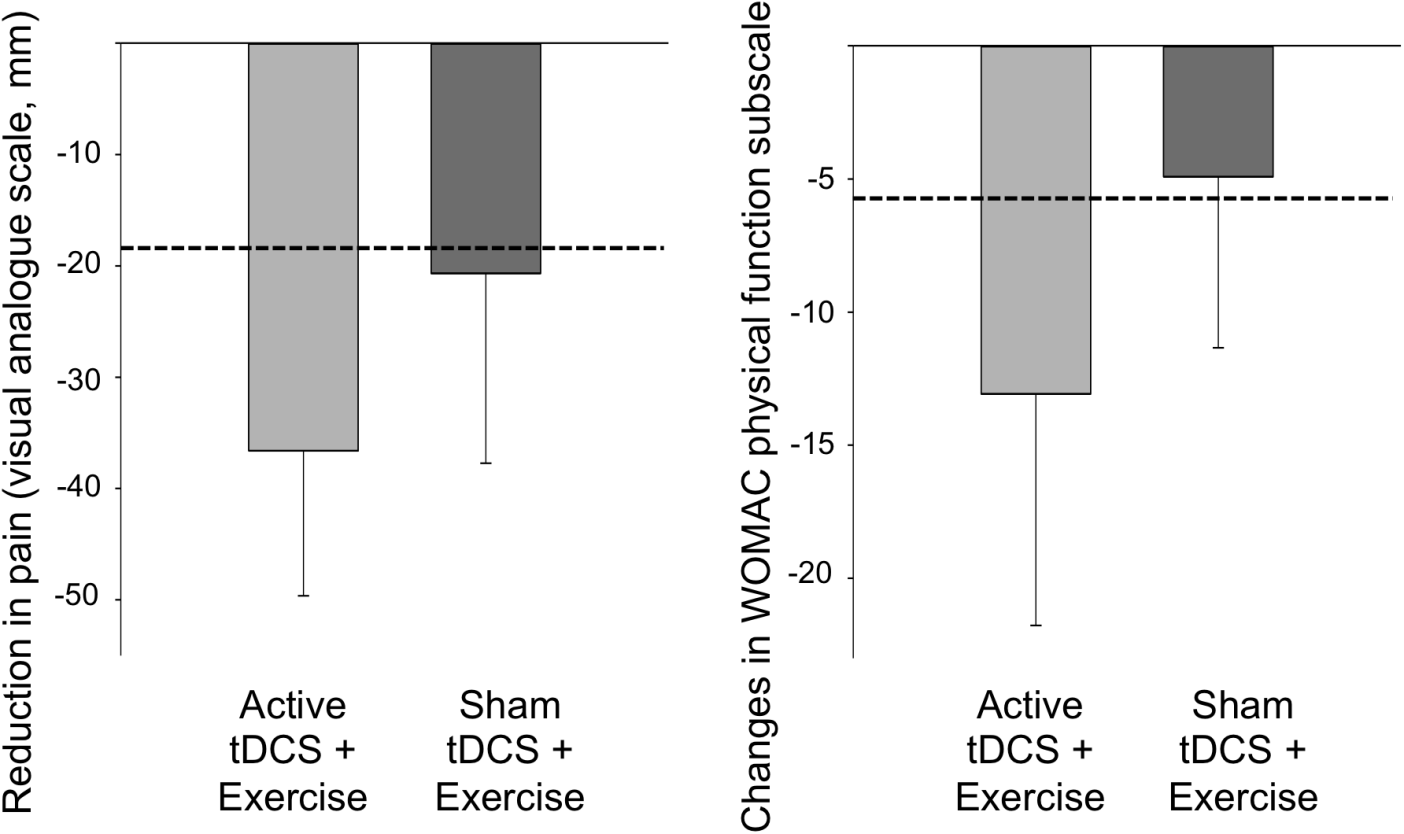
Group change in pain (left panel) and WOMAC physical function subscale (right panel). The graph showed within-group changes (mean and 95% confidence interval) in pain and function following 8 weeks of either active tDCS + exercise or sham tDCS + exercise. Note: larger negative scores indicate greater improvements in pain and function. The dotted line indicates the minimal clinically important change for each outcome.

Pain during walking (100 mm VAS) reduced in both groups at post-intervention (ANOVA: AT+EX group: p<0.001, partial η^2^ = 0.55; ST+EX group: p = 0.026, partial η^2^ = 0.18) (Table 2) (Fig 3). Pain reduction in the AT+EX group was double that observed in the ST+EX group (AT+EX group: -41.4 mm, 95%CI -30.7 to -52.2; ST+EX group: -20.7 mm, 95%CI -7.1 to -34.3; Fig 4). The between-group difference was in favour of the AT+EX group (mean difference = -13.0, 95%CI -32.6 to 6.5; ANCOVA: p = 0.18, partial η^2^ = 0.08). Scores on the WOMAC pain subscale followed a similar pattern (Table 2).

**Table 2:** Group data (mean and 95% confidence interval) for pain and function outcome measures.

	Baseline		Post-intervention		Difference within groups (Follow up–Baseline) ^a^		Difference between groups; adjusted mean ^b^	
	AT+EX (N = 15)	ST+EX (N = 15)	AT+EX (N = 13)	ST+EX (N = 12)	AT+EX (N = 13)	ST+EX (N = 12)	AT+EX minus ST+EX	P value between groups
**Pain VAS (100 mm)**	59.9(67.6,52.1)	56.5(66.5,46.5)	24.1(33.4,14.8)*	33.7(49.0,18.5)*	-41.4(-30.7,-52.2)	-20.7(-7.1,-34.3)	-13.0(-32.6,6.5)	.18
**WOMAC**								
**Total score**	55.0(63.1,46.9)	48.0(53.4,42.6)	36.8(45.3,28.2)*	39.1(47.1,31.0)	-16.7(-6.0,-27.3)	-8.1(-1.3,-14.8)	-6.2(-18.8,6.3)	.31
**Pain subscale**	11.0(13.0,9.0)	9.9(11.6,8.3)	7.5(9.2,5.7)*	7.4(9.3,5.5)	-3.8(-1.0,-6.5)	-2.2(-0.5,-3.8)	-0.6(-3.4,2.3)	.69
**Physical function subscale**	38.9(44.9,32.8)	33.3(37.2,29.3)	26.0(32.3,19.7)*	27.8(33.8,21.7)	-10.9(-3.3,-18.5)	-4.9(0.2,-10.0)	-4.8(-14.0,4.3)	.28

AT + EX = active tDCS + exercise, ST + EX = sham tDCS + exercise; VAS = visual analogue scale, WOMAC = Western Ontario and McMaster Universities Osteoarthritis Index.

^a^ A negative number indicates improvement at post-intervention.

^b^ A negative number favours the AT + EX group.

*p < 0.05.

Improvements in physical function (WOMAC subscale) were observed in the AT+EX (ANOVA: p = 0.01, partial η^2^ = 0.22), but not the ST+EX group (ANOVA: p = 0.16, partial η^2^ = 0.08) at post-intervention (AT+EX: -10.9 units, 95%CI -3.3 to -18.5; ST+EX: -4.91 units, 95%CI 0.2 to -10.0; Figs 3 and 4). Between-group comparisons did not reach statistical significance (mean difference = -4.8, 95%CI -14.0 to 4.3; ANCOVA: p = 0.28, partial η^2^ = 0.052).

### Pain mechanisms

**Fig 5:**
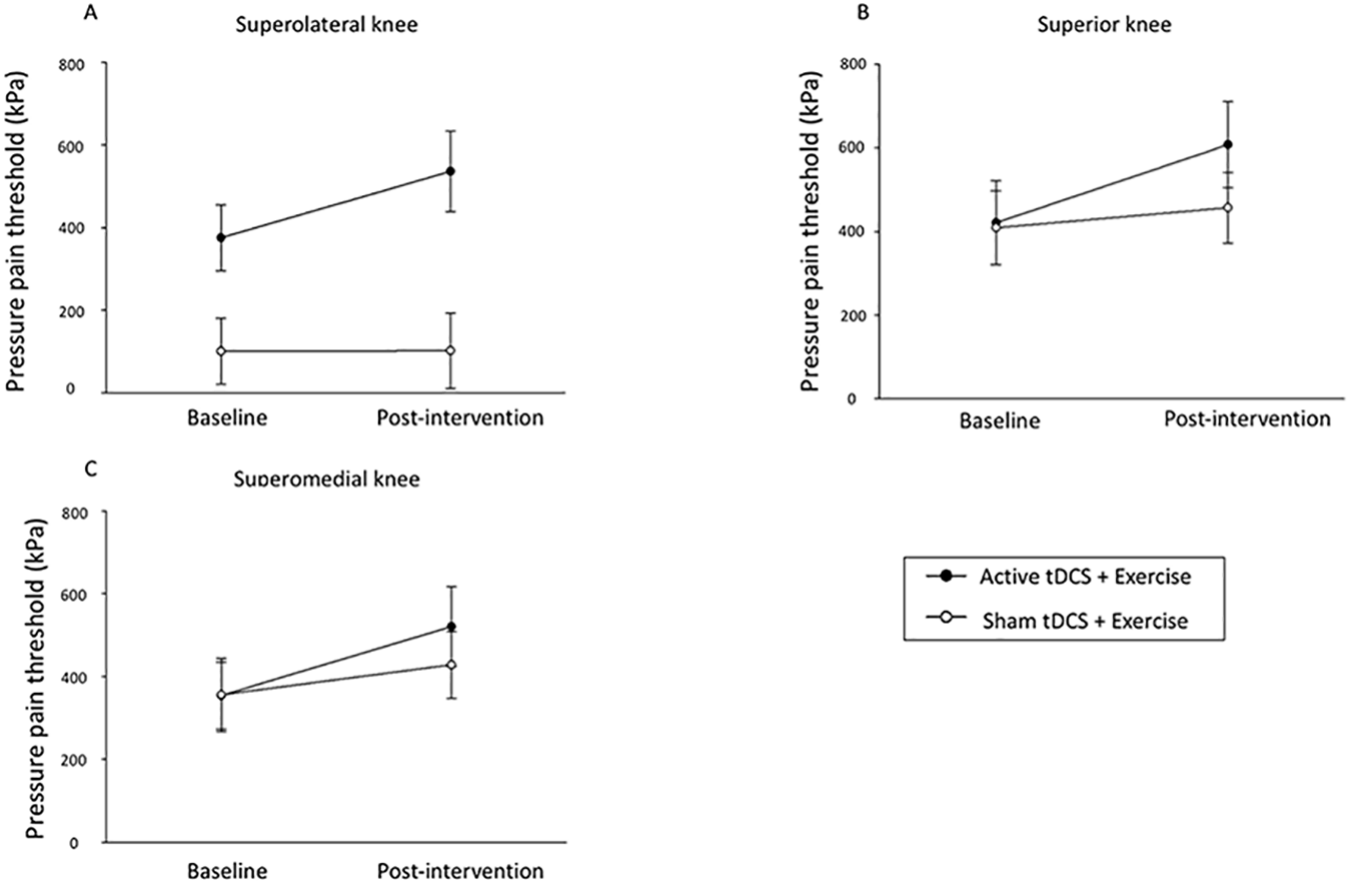
Pressure pain thresholds (mean and 95% confidence interval) pre- and post-interventions at three knee sites. Active tDCS + exercise produced greater improvements in pressure pain thresholds at all three sites following 8 weeks of treatment compared with sham tDCS + exercise (A = superolateral knee; B = superior knee; C = superomedial knee).

PPTs increased (i.e. a greater amount of pressure was required to be perceived as painful) to a greater extent in the AT+EX group for the superolateral, superior, and superomedial knee sites when compared with the ST+EX group (ANCOVA: p<0.05; partial η^2^ = 0.169, partial η^2^ = 0.301, partial η^2^ = 0.262 respectively) (Fig 5). Conditioned pain modulation, which is proposed to measure descending pain inhibition (TS at the worst knee and CS at the contralateral forearm), improved from baseline in the AT+EX group (25.6 kPa, 95%CI 47.2 to 4.1, ANOVA: p = 0.032, partial η^2^ = 0.17) but not in the ST+EX group (-27.1 kPa, 95%CI 24.6 to -78.8, ANOVA: p = 0.41, partial η^2^ = 0.03) (see Supplementary S1 Table). However, there were no between-group differences (mean difference = 39.0, 95%CI -0.7, 78.6; ANCOVA: p = 0.054, partial η^2^ = 0.158). No within- or between-group differences were found for any other measure, including the NFR (Supplementary S1, S2 and S3 Tables).

### Sample size calculation

The minimum clinically important difference to be detected in OA trials is a change in pain of 18 mm and a change in function of six units [42]. We require a sample size of 99 participants per intervention arm (198 in total) at 90% power and 5% significance level to detect a mean difference of this magnitude, assuming a small effect size (0.3) and allowing for a maximum dropout rate of 20%.

## Discussion

This is the first study to investigate the addition of tDCS to a quadriceps strengthening exercise in knee OA. Our study demonstrates that this treatment combination is feasible and appears to be safe in this population. Further, our preliminary evidence indicates that adding tDCS to exercise may be a promising approach for improving pain, physical function and pain mechanisms in knee OA. These results provide data to inform a fully powered clinical trial to examine the effect of this novel treatment on the symptoms and pain mechanisms associated with knee OA.

Attendance rates for treatments and post-intervention assessment were both above 80%, indicating that a larger randomised controlled trial to evaluate the efficacy of this treatment in this population is feasible [43]. No barriers to implementation of the interventions or outcome measures were identified in this study. Therefore, the methodology used in this study can be implemented in a larger study without any major amendments. Adverse reactions to tDCS during (e.g. fatigue) and after (headache, nausea or insomnia) stimulation have been reported in previous studies [21, 44, 45]. We documented only two adverse reactions that could be attributed to tDCS: one episode of headache in the AT+EX group and one episode of a painful sensation during the initial ramping up of the electric current in the ST+EX group. No adverse reactions were documented in response to the exercise treatment. The overall incidence rate of adverse reactions in this study is lower than those reported in either the tDCS or knee OA literature [44, 46], indicating that the implementation of a tDCS and exercise treatment is likely to be safe in knee OA.

Previous studies have investigated the analgesic effect of tDCS in chronic pain conditions such as low back pain [15, 47], chronic pelvic pain [18], fibromyalgia [21, 48, 49] and neuropathic pain after spinal cord injury [50] with conflicting results. This study is the first to use tDCS in knee OA and to combine tDCS with strengthening exercise in any pain condition. Consistent with evidence of strengthening exercise in knee OA [5, 51], both groups reported reduced pain following the 8-week treatment. However, in the AT+EX group, effects on pain were more than double the minimal clinically important difference (MCID) of 20 mm for this outcome [42], and double those observed in the ST+EX group. The improvement in physical function following AT+EX also exceeded the MCID of 6 units on the WOMAC physical function subscale in knee OA [42].

Sensitivity to pressure was reduced to a greater extent (increase in PPTs) following AT+EX than ST+EX. CPM (presumed to indicate descending pain inhibition) also demonstrated similar results. The potentially superior effects on pain system function observed with AT+EX may reflect a summative effect of the two treatments on pain mechanisms. Pain in knee OA is considered to include contributions from both peripheral nociceptive afferents in the knee joint structures, as well as sensitization, both peripherally and centrally, and the relative contribution of each will vary between individuals. Recognition of the role of central sensitisation in knee OA is increasing. From one perspective, persistent nociceptive input from joint structural changes in knee OA can increase the synaptic excitability and efficiency in the central pain pathway and result in central sensitisation, characterised by local and widespread hyperalgesia [52, 53], augmented spinal excitability and deficits in descending pain inhibition [54, 55]. Multiple other factors contribute to this process including unhealthy pain cognitions and a host of biological processes. Pain intensity in many individuals with knee OA is associated with hyperalgesia and impaired descending pain inhibition, and for many the relationship with radiographic changes is weak [56]. Exercise is known to have an anti-nociceptive effect at both peripheral and central levels [20, 57–59], and the potential to reduce the “pain” sensitivity in the central nervous system, in chronic pain conditions [60]. Anodal tDCS can modulate pain processing at central level [16] and increase the brain’s receptiveness to other interventions through a ‘priming’ effect by modulating the excitability of cortical neurons/networks [61]. Adding anodal tDCS to exercise may induce complementary effects on pain mechanisms and bolster the brain’s responsiveness to the analgesic effects of exercise, leading to greater clinical benefits in knee OA. The relationship between a tDCS and exercise treatment, pain mechanisms and clinical benefits requires investigation in a larger randomised controlled trial.

An alternative explanation for our findings is that tDCS primed/enhanced the corticomotor training effects of strengthening exercise. Previous studies of tDCS combined with strength training in healthy individuals have shown a greater capacity for high volume training, lower perceived exertion during training, improved motor control and larger increases in corticomotor excitability than can be achieved with strength training alone [62, 63]. These effects may lead to greater improvements in knee joint control and mechanical benefits for the knee, reducing pain and disability. Future studies should include measures of muscle strength and motor control to further evaluate this possibility.

tDCS is a relatively inexpensive and portable device and for health professionals already trained in the therapeutic use of electric current, such as physiotherapists, minimal training would be required to ensure safe and effective application. Although not currently used in the clinical setting, tDCS could be easily integrated into clinical practice if beneficial effects on knee OA are established in a future larger trial. A fully powered randomised controlled trial is required to determine whether this treatment produces superior clinical benefits in knee OA.

This study had several limitations. First, by design the study included a small sample size that was not intended to provide sufficient power to definitively determine the efficacy of adding tDCS to exercise treatment for knee OA. Therefore, the results must be interpreted with caution. Second, the short follow-up period in this study may have been too brief to determine between-group differences. A larger clinical trial with longer follow-up periods is required. Third, we did not record any changes in the participants’ medication (type and dosage) during this trial. The dosage of the participants’ usual medication was only recorded at the baseline. Future trials should record any changes in participants’ use of medication during the trial to evaluate the relationship between pain and the use of medication. Finally, the treating physiotherapists delivered both the tDCS and exercise treatment, and were not blind to group allocation. However, our exercise protocol was well established with clear instructions for how to progress each exercise [23] and the treating therapists were instructed to strictly adhere to the protocol to minimise any potential bias. Future trials should seek to blind the treating therapists to the tDCS condition.

This pilot study provides the first feasibility and safety data for the addition of tDCS to strengthening exercise in people with knee OA. Although not powered to assess between-group differences, our study suggests that the addition of active tDCS to exercise may improve pain, function and pain mechanisms in knee OA beyond that of sham tDCS with exercise, and in excess of MCIDs for pain and function in this population. A fully powered randomised controlled trial with longer follow up is now justified to determine the clinical benefit of this novel treatment for knee OA.

## Supporting information

S1 Table Group data (mean and 95% confidence interval) for heat pain thresholds, conditioned pain modulation and nociceptive withdraw reflex. AT+EX = active tDCS + exercise, ST+EX = sham tDCS + exercise, HPT = heat pain threshold, CPM = conditioned pain modulation, NFR = nociception flexor withdraw reflex, RMS = root mean square.
(DOCX)

S2 Table Group data (mean and 95% confidence interval) for pressure pain thresholds. AT+EX = active tDCS + exercise, ST+EX = sham tDCS + exercise; Knee 1 = 2 cm distal to the inferior medial edge of patella, Knee 2 = 2 cm distal to the interior lateral edge of patella, Knee 3 = 3 cm lateral the mid point of the lateral patellar border, Knee 4 = 2 cm proximal to the superior lateral edge of patella, Knee 5 = 2 cm proximal to the mid point of the superior patellar border, Knee 6 = 2 cm medial to the superior medial edge of patellar, Knee 7 = medial to the mid point of the medial patellar border, Knee 8 = centre of the patella.
(DOCX)

S3 Table Effect size (Cohen’s *d*) of difference within groups for pain, function and pain mechanisms. WOMAC = Western Ontario and McMaster Universities Osteoarthritis Index; Knee 1 = 2 cm distal to the inferior medial edge of patella, Knee 2 = 2 cm distal to the interior lateral edge of patella, Knee 3 = 3 cm lateral the mid point of the lateral patellar border, Knee 4 = 2 cm proximal to the superior lateral edge of patella, Knee 5 = 2 cm proximal to the mid point of the superior patellar border, Knee 6 = 2 cm medial to the superior medial edge of patellar, Knee 7 = medial to the mid point of the medial patellar border, Knee 8 = centre of the patella; RMS = root mean square.
(DOCX)

S4 Table CONSORT 2010 checklist of information to include when reporting a randomised trial. (DOC)

S1 File Protocol paper manuscript. (PDF)
