# Supplementary material for: Addition of transcranial direct current stimulation to quadriceps strengthening exercise in knee osteoarthritis: A pilot randomised controlled trial
Source: PLoS One. 2017 Jun 30;12(6):e0180328. doi: 10.1371/journal.pone.0180328 (PMC5493377; doi:10.1371/journal.pone.0180328)
Supplement: S1 Table — AT+EX = active tDCS + exercise, ST+EX = sham tDCS + exercise, HPT = heat pain threshold, CPM = conditioned pain modulation, NFR = nociception flexor withdraw reflex, RMS = root mean square. (DOCX) [file pone.0180328.s001.docx]

**S1 Table.** **Group data (mean and 95% confidence interval) for heat pain thresholds, conditioned pain modulation and nociceptive flexor withdraw reflex.**

|  | | **Baseline** | | **Follow-up** | | **Difference within groups**  **(Follow up – Baseline)** | | **Difference between groups;**  **adjusted mean^a^** | |
| --- | --- | --- | --- | --- | --- | --- | --- | --- | --- |
|  |  | **AT+EX**  **(N = 15)** | **ST+EX**  **(N = 15)** | **AT+EX**  **(N = 13)** | **ST+EX**  **(N = 12)** | **AT+EX**  **(N = 13)** | **ST+EX**  **(N = 12)** | **AT+EX minus**  **ST+EX** | **P value between groups** |
| **HPT (°C)** | **Medial knee** | 44.8 (45.8, 43.8) | 44.9 (46.2, 43.5) | 45.3 (46.0, 44.6) | 45.1 (46.1, 44.1) | 0.3 (0.9, -0.3) | 0.9 (2.1, -0.3) | -0.2 (-1.4, 1.0) | .58 |
| **Anterior**  **knee** | | 44.2 (45.5, 42.8) | 44.8 (45.9, 43.7) | 44.6 (45.8, 43.4) | 44.7 (46.4, 43.0) | -0.6 (1, -2.2) | 0.2 (1.8, -1.3) | -0.2 (-2.5, 2.0 | .82 |
| **Lateral**  **knee** | | 45.5 (46.5, 44.5) | 46.2 (47.0, 45.4) | 45.7 (46.5, 44.9) | 46.5 (47.5, 45.5) | -0.1 (0.4, -0.6) | 0.6 (1.6, -0.5) | -0.8 (-2.1, 0.6) | .24 |
| **Ipsilateral**  **forearm** | | 42.6 (44.2, 41.0) | 43.7 (44.9, 42.5) | 44.4 (45.5, 44.4) | 44.5 (45.3, 43.8) | 1.7 (2.7, 0.7) | 1.2 (2.3, 0.1) | -0.8 (-2.2, 0.6) | .67 |
| **Contralateral forearm** | | 43.4 (44.8, 42.0) | 43.1 (44.4, 41.8) | 44.6 (45.9, 43.2) | 44.7 (45.5, 43.9) | 1.4 (2.3, 0.4) | 2.0 (3.5, 0.4) | -0.5 (-2.2, 1.1) | .79 |
| **CPM (kPa)** | **Knee/**  **Arm** | 44.2 (72.6, 15.7) | 73.8 (115.1, 32.34) | 88.1 (110.6, 65.6) * | 51.7 (75.4, 28.0) | 25.7 (47.2, 4.1) | -27.1 (24.6, -78.8) | 39.0 (-0.7, 78.6) | .054 |
| **Arm/Arm** | | 18.3 (43.1, -6.3) | 26.5 (53.9, -0.8) | 61.3 (94.2, 28.4) | 46.9 (73.4, 20.5) | 19.6 (31.9, 7.2) | 21.1 (56.3, -14.1) | 3.5 (-36.5, 43.4) | .85 |
| **NFR** | **Threshold (mA)** | 135.4 (184.3, 86.6) | 102.7 (144.6, 60.8) | 167.0 (229.1, 104.9) | 132.9 (180.6, 85.2) | 13.0 (37.5, -11.5) | 13.4 (50.4, -23.6) | 8.4 (-46.3, 63.2) | .75 |
| **Latency (ms)** | | 125.2 (135.4, 115.1) | 122.5 (135.2, 109.8) | 130.6 (143.1, 118.0) | 116.5 (128.4, 104.5) | 10.9 (24.2, -2.4) | -8.9 (2.4, -20.1) | 14.6 (-3.9, 33.1) | .11 |
| **Amplitude (RMS)** | | 0.12 (0.19, 0.05) | 0.15 (0.24, 0.06) | 0.08 (0.1, 0.07) | 0.1 (0.15, 0.06) | -0.06 (0.02, -0.14) | -0.04 (0.03, -0.12) | -0.01 (-0.07, 0.04) | .56 |

AT+EX = active tDCS + exercise, ST+EX = sham tDCS + exercise, HPT = heat pain threshold, CPM = conditioned pain modulation, NFR = nociception flexor withdraw reflex, RMS = root mean square. ^a^ Value adjusted for baseline scores using ANCOVA. * Indicates statistically significant (*p*<0.05) improvement from baseline within each treatment group.
