# Supplementary material for: Addition of transcranial direct current stimulation to quadriceps strengthening exercise in knee osteoarthritis: A pilot randomised controlled trial
Source: PLoS One. 2017 Jun 30;12(6):e0180328. doi: 10.1371/journal.pone.0180328 (PMC5493377; doi:10.1371/journal.pone.0180328)
Supplement: S2 Table — AT+EX = active tDCS + exercise, ST+EX = sham tDCS + exercise; Knee 1 = 2 cm distal to the inferior medial edge of patella, Knee 2 = 2 cm distal to the interior lateral edge of patella, Knee 3 = 3 cm lateral the mid point of the lateral patellar border, Knee 4 = 2 cm proximal to the superior lateral edge of patella, Knee 5 = 2 cm proximal to the mid point of the superior patellar border, Knee 6 = 2 cm medial to the superior medial edge of patellar, Knee 7 = medial to the mid point of the medial patellar border, Knee 8 = centre of the patella. (DOCX) [file pone.0180328.s002.docx]

**S2 Table.** **Group data (mean and 95% confidence interval) for pressure pain thresholds.**

|  | **Baseline** | | **Follow-up** | | **Difference within groups**  **(Follow up – Baseline)** | | **Difference between groups;**  **adjusted mean^a^** | |
| --- | --- | --- | --- | --- | --- | --- | --- | --- |
|  | **AT+EX**  **(N = 15)** | **ST+EX**  **(N = 15)** | **AT+EX**  **(N = 13)** | **ST+EX**  **(N = 12)** | **AT+EX**  **(N = 13)** | **ST+EX**  **(N = 12)** | **AT+EX minus**  **ST+EX** | **P value between groups** |
| **Ipsilateral forearm** | 345.7 (424.0, 267.4) | 294.2 (347.4, 240.9) | 445.8 (535.0, 356.7) | 335.7 (386.1, 285.4) | 78.2 (191.1, -34.6) | 52.4 (116.1, -11.3) | 85.2 (352.7, 515.2) | .15 |
| **Ipsilateral tibialis anterior** | 349.2 (427.6, 270.9) | 369.1 (453.3, 285.0) | 500.9 (557.4, 444.4) ** | 441.7 (496.5, 386.8) | 116.5 (230, 2.9) | 80.3 (148.4, 122.0) | 60.2 (-27.4, 148.0) | .16 |
| **Knee 1** | 451.5 (568.0, 334.9) | 473.5 (587.4, 359.6) | 612.8 (723.4, 502.2) | 560.6 (658.8, 462.4) | 126.1 (183.5, 68.9) | 751.2 (182.4, -32.1) | 55.8 (-60.7, 172.3) | .33 |
| **Knee 2** | 409.1 (507.5, 310.7) | 429.3 (517.5, 341.0) | 611.5 (718.5, 504.5) ** | 578.4 (672.1, 484.8) ** | 196.1 (279.6, 112.7) | 165.7 (283.5, 47.9) | 25.6 (-117.2, 168.4) | .71 |
| **Knee 3** | 344.6 (420.4, 268.66) | 338.0 (388.3, 287.7) | 499.8 (573.2, 426.4) ** | 444.6 (524.3, 365.0) ** | 155.7 (222.8, 88.7) | 96.5 (157.0, 36.1) | 49.3 (-45.7, 144.4) | .29 |
| **Knee 4** | 375.9 (449.1, 302.7) | 340.9 (414.3, 267.5) | 536.8 (618.4, 455.2) ** | 409.3 (481.8, 336.9) | 192.9 (244.1, 141.7) | 82.1 (139.0, 25.3) | 110.2 (4.8, 215.7) | .041* |
| **Knee 5** | 421.8 (513.8, 329.8) | 409.6 (490.0, 329.2) | 608.3 (693.9, 522.7) ** | 457.7 (525.0, 390.4) | 194.4 (258.7, 130.2) | 52.6 (106.0, -0.7) | 164.8 (56.5, 273.1) | .005* |
| **Knee 6** | 353.9 (428.0, 279.8) | 355.7 (436.5, 275.0) | 520.5 (601.0, 439.9) ** | 428.2 (492.5, 363.8) | 178.1 (232.8, 123.4) | 59.9 (108.7, 11.3) | 123. 5 (34.1, 212.9) | .009* |
| **Knee 7** | 311.1 (370.1, 252.0) | 326.9 (385.4, 268.4) | 466.3 (535.4, 397.2) ** | 448.1 (489.9, 406.3) ** | 159.1 (198.9, 119.3) | 110.8 (146.0, 75.7) | 58.1 (-19.0, 135.1) | .13 |
| **Knee 8** | 384.2 (455.0, 313.2) | 388.3 (463.0, 313.5) | 538.5 (620.0, 457.0) ** | 488.0 (583.8, 392.1) | 160.9 (216.1, 105.7) | 102.5 (199.1, 59.1) | 40.4 (-84.4, 165.1) | .50 |

AT+EX = active tDCS + exercise, ST+EX = sham tDCS + exercise; Knee 1 = 2 cm distal to the inferior medial edge of patella, Knee 2 = 2 cm distal to the interior lateral edge of patella, Knee 3 = 3 cm lateral the mid point of the lateral patellar border, Knee 4 = 2 cm proximal to the superior lateral edge of patella, Knee 5 = 2 cm proximal to the mid point of the superior patellar border, Knee 6 = 2 cm medial to the superior medial edge of patellar, Knee 7 = medial to the mid point of the medial patellar border, Knee 8 = centre of the patella. * Between group P < 0.05. ^a^ Value adjusted for baseline scores using ANCOVA. ** Indicates statistically significant (*p*<0.05) improvement from baseline within each treatment group.
