# Supplementary material for: Addition of transcranial direct current stimulation to quadriceps strengthening exercise in knee osteoarthritis: A pilot randomised controlled trial
Source: PLoS One. 2017 Jun 30;12(6):e0180328. doi: 10.1371/journal.pone.0180328 (PMC5493377; doi:10.1371/journal.pone.0180328)
Supplement: S3 Table — WOMAC = Western Ontario and McMaster Universities Osteoarthritis Index; Knee 1 = 2 cm distal to the inferior medial edge of patella, Knee 2 = 2 cm distal to the interior lateral edge of patella, Knee 3 = 3 cm lateral the mid point of the lateral patellar border, Knee 4 = 2 cm proximal to the superior lateral edge of patella, Knee 5 = 2 cm proximal to the mid point of the superior patellar border, Knee 6 = 2 cm medial to the superior medial edge of patellar, Knee 7 = medial to the mid point of the medial patellar border, Knee 8 = centre of the patella; RMS = root mean square. (DOCX) [file pone.0180328.s003.docx]

**S3 Table.** **Effect size (Cohen’s *d*) of difference within groups for pain, function and pain mechanisms.**

|  |  | **Active tDCS + Eexercise** | **Sham tDCS + Exercise** |
| --- | --- | --- | --- |
| **Pain (Visual analogue scale)** | | -1.95 | -0.77 |
| **WOMAC** | **Total score** | -0.79 | -0.61 |
|  | **Pain subscale** | -0.70 | -0.66 |
|  | **Physical function subscale** | -0.73 | -0.49 |
| **Heat pain threshold** | **Medial knee** | 0.28 | 0.39 |
|  | **Anterior knee** | -0.18 | 0.08 |
|  | **Lateral knee** | -0.10 | 0.27 |
|  | **Ipsilateral forearm** | 0.88 | 0.56 |
|  | **Contralateral forearm** | 0.70 | 0.65 |
| **Pressure pain threshold** | **Knee 1** | 1.36 | 0.32 |
|  | **Knee 2** | 1.32 | 0.61 |
|  | **Knee 3** | 1.30 | 0.68 |
|  | **Knee 4** | 1.44 | 0.73 |
|  | **Knee 5** | 1.30 | 0.50 |
|  | **Knee 6** | 1.61 | 0.62 |
|  | **Knee 7** | 2.06 | 1.60 |
|  | **Knee 8** | 1.18 | 0.54 |
|  | **Ipsilateral tibialis anterior** | 0.49 | 0.37 |
|  | **Ipsilateral forearm** | 0.75 | 0.52 |
| **Nociception flexor withdraw reflex** | **Threshold** | 0.27 | 0.18 |
|  | **Latency** | 0.42 | -0.40 |
|  | **RMS** | -0.38 | -0.28 |
| **Conditioned pain modulation** | **Knee/Arm** | 0.77 | -0.27 |
|  | **Arm/Arm** | 0.86 | 0.30 |

WOMAC = Western Ontario and McMaster Universities Osteoarthritis Index; Knee 1 = 2 cm distal to the inferior medial edge of patella, Knee 2 = 2 cm distal to the interior lateral edge of patella, Knee 3 = 3 cm lateral the mid point of the lateral patellar border, Knee 4 = 2 cm proximal to the superior lateral edge of patella, Knee 5 = 2 cm proximal to the mid point of the superior patellar border, Knee 6 = 2 cm medial to the superior medial edge of patellar, Knee 7 = medial to the mid point of the medial patellar border, Knee 8 = centre of the patella; RMS = root mean square.
