# Supplementary material for: Addition of transcranial direct current stimulation to quadriceps strengthening exercise in knee osteoarthritis: A pilot randomised controlled trial
Source: PLoS One. 2017 Jun 30;12(6):e0180328. doi: 10.1371/journal.pone.0180328 (PMC5493377; doi:10.1371/journal.pone.0180328)
Supplement: S1 File — (PDF) [file pone.0180328.s005.pdf]

National Ethics Application Form
Version 2008 - V2.0

Proposal title: **Pain, physiotherapy and underlying cortical mechanisms**

For submission to:
University of Western Sydney Human Research Ethics Committee (EC00314)

Name: **Prof Lucinda Chipchase**

Address: **University of Western Sydney**
School of Science and Health
Locked Bag 1797
Penrith NSW 2751
Australia

Contact: (Bus) **0412133210**
(AH) **-**
(Mob) **0412133210**
(Fax) **02 4620 3710**

Proposal status: **Complete**

Proposal description:

Persistent musculoskeletal pain is one of the most significant health issues in the developed world, with an economic burden second only to cancer (Lim et al., 2013). Despite the enormity of the problem, many current therapies target generic symptoms, not underlying mechanisms and show, at best, small effects (Airaksinen et al., 2004). In 2010, the Australian National Pain Summit concluded 'the management of pain is shockingly inadequate'. This assessment is not surprising given that critical information on the mechanisms that underpin persistent pain is lacking.

Evidence suggests that persistent pain resides in the brain. Mechanisms such as increased sensitivity of the brain and spinal cord (known as central sensitisation) and abnormal organisation of key brain regions are now widely believed to explain why some people recover after musculoskeletal injury and others do not. Despite this belief, few studies have investigated these mechanisms in acute and persistent pain states. If these mechanisms can be comprehensively linked to persistent pain, new therapies that target these mechanisms can be developed that improve outcomes after musculoskeletal injury.

The overall aims of this program of research are to:

1) To understand the role of the brain and spinal cord in acute and persistent musculoskeletal pain.

2) To test novel therapies that target these mechanisms in musculoskeletal pain.

This program of research involves a series of experimental laboratory based studies with participants in two groups:

Group 1 will include either healthy individuals who will receive an injection of hypertonic saline to induce acute muscle pain, or individuals experiencing persistent musculoskeletal pain.

Group 2 includes age and gender matched healthy control subjects who are pain free.

Laboratory measures include investigation of brain connectivity, spinal cord excitability, peripheral nerve excitability, muscle activity, pressure and thermal pain threshold tests and the induction of acute muscle pain. Clinical interventions will include non-invasive brain stimulation, electrical stimulation of nerves and exercise. Participants will also complete questionnaires about their pain, function and psychosocial well-being.

Note, this application is a program ethics application.

Previously submitted to:
The University of Queensland Medical Research Ethics Committee (EC00179)

Administrative Section

1. TITLE AND SUMMARY OF PROJECT

1.1. Title

1.1.1 What is the formal title of this research proposal?

Pain, physiotherapy and underlying cortical mechanisms

1.1.2 What is the short title / acronym of this research proposal (if applicable)?

Understanding pain

1.2. Description of the project in plain language

1.2.1 Give a concise and simple description (not more than 400 words), in plain language, of the aims of this project, the proposal research design and the methods to be used to achieve those aims.

Persistent musculoskeletal pain is one of the most significant health issues in the developed world, with an economic burden second only to cancer (Lim et al., 2013). Despite the enormity of the problem, many current therapies target generic symptoms, not underlying mechanisms and show, at best, small effects (Airaksinen et al., 2004). In 2010, the Australian National Pain Summit concluded 'the management of pain is shockingly inadequate'. This assessment is not surprising given that critical information on the mechanisms that underpin persistent pain is lacking.

Evidence suggests that persistent pain resides in the brain. Mechanisms such as increased sensitivity of the brain and spinal cord (known as central sensitisation) and abnormal organisation of key brain regions are now widely believed to explain why some people recover after musculoskeletal injury and others do not. Despite this belief, few studies have investigated these mechanisms in acute and persistent pain states. If these mechanisms can be comprehensively linked to persistent pain, new therapies that target these mechanisms can be developed that improve outcomes after musculoskeletal injury.

The overall aims of this program of research are to:

1) To understand the role of the brain and spinal cord in acute and persistent musculoskeletal pain.

2) To test novel therapies that target these mechanisms in musculoskeletal pain.

This program of research involves a series of experimental laboratory based studies with participants in two groups:

Group 1 will include either healthy individuals who will receive an injection of hypertonic saline to induce acute muscle pain, or individuals experiencing persistent musculoskeletal pain.

Group 2 includes age and gender matched healthy control subjects who are pain free.

Laboratory measures include investigation of brain connectivity, spinal cord excitability, peripheral nerve excitability, muscle activity, pressure and thermal pain threshold tests and the induction of acute muscle pain. Clinical interventions will include non-invasive brain stimulation, electrical stimulation of nerves and exercise. Participants will also complete questionnaires about their pain, function and psychosocial well-being.

Note, this application is a program ethics application.

2. RESEARCHERS / INVESTIGATORS

2.1. Chief researcher(s) / investigator(s)

2.2. Principal researcher(s) / investigator(s)

2.2.0 How many principal researchers / investigators are there? 4

2.2.1. Principal researcher / investigator 1

2.2.1. Name and contact details

Name: Prof Lucinda Chipchase

Address: University of Western Sydney
School of Science and Health
Locked Bag 1797
Penrith NSW 2751
Australia

Organisation: University of Western Sydney

Area: School of Science and Health

Position: Professor of Physiotherapy

Contact (Bus) 0412133210 (AH) -
(Mob) 0412133210 (Fax) 02 4620 3710

Email: l.chipchase@uws.edu.au

2.2.2... Summary of qualifications and relevant expertise NS 4.8.7 NS 4.8.15

Dr Chipchase has entry level qualifications in physiotherapy, a Masters of Musculoskeletal Physiotherapy and a PhD in physiotherapy. She has experience in a broad range of research methodologies including randomised controlled trials, clinical trials, observational approaches including surveys and Delphi questionnaires. In addition, her clinical and theoretical knowledge combined with her hypotheses on the effect of clinical interventions at the cortex are central to this application.

She has expertise in neurophysiological research techniques outlined in the current proposal including transcranial magnetic stimulation to probe the motor system; induced pain methodologies, electromyography and electroencephalography to measure sensory evoked potentials. Her research in this area has resulted in several publications in high impact neuroscience and physiotherapy journals.

2.2.2... Please declare any general competing interests

No competing interests

2.2.2... Name the site(s) for which this principal researcher / investigator is responsible.

University of Western Sydney

2.2.3 Describe the role of the principal researcher / investigator in this project.

Chief investigator. Leads the design and development of the program of research.

2.2.4 Is the principal researcher / investigator a student? No

2.2.1. Principal researcher / investigator 2

2.2.1. Name and contact details

Name: Dr Siobhan Schabrun

Address: University of Queensland
School of Health and Rehabilitation Sciences
St Lucia QLD 4072

Organisation: The University of Queensland

Area: School of Health and Rehabilitation Sciences

Position: NHMRC Post Doctoral Fellow

Contact (Bus) 07 3365 4590 (AH) -

(Mob) - (Fax) -

Email: s.schabrun@uq.edu.au

2.2.2... Summary of qualifications and relevant expertise NS 4.8.7 NS 4.8.15

Dr Schabrun is an NHMRC post doctoral fellow with a focus on cortical plasticity, non-invasive brain stimulation and neurorehabilitation. She has a doctorate in neurophysiology and an undergraduate honours degree in physiotherapy providing her with strong skills in both basic and clinical research. Her current post-doctoral research (funded by a NHMRC Clinical Research Training Fellowship) extends her work in the field of plasticity into the development and testing of novel rehabilitation strategies. The focus of the current proposal fits directly within this theme.

2.2.2... Please declare any general competing interests

There are no competing interests.

2.2.2... Name the site(s) for which this principal researcher / investigator is responsible.

The University of Queensland

2.2.3 Describe the role of the principal researcher / investigator in this project.

Contribute to the development and design of experiments, the analysis and write up of publications.

2.2.4 Is the principal researcher / investigator a student? No

2.2.1. Principal researcher / investigator 3

2.2.1. Name and contact details

Name: Ms Emma Jones

Address: University of Western Sydney
School of Science and Health
Locked Bag 1797
Penrith NSW 2751

Organisation: University of Western Sydney

Area: School of Science and Health

Position: PhD student

Contact (Bus) 02 4620 3758 (AH) -
(Mob) - (Fax) -

Email: 17614317@student.uws.edu.au

2.2.2... Summary of qualifications and relevant expertise NS 4.8.7 NS 4.8.15

Bachelor of Biomedical Science, University of Newcastle
3 years experience as a Research Technician at the CCRE Spine at the University of Queensland using the techniques outlined in this proposal.

2.2.2... Please declare any general competing interests

There are no competing interests.

2.2.2... Name the site(s) for which this principal researcher / investigator is responsible.

University of Western Sydney

2.2.3 Describe the role of the principal researcher / investigator in this project.

Phd Student

2.2.4 Is the principal researcher / investigator a student? Yes

2.2.4...What is the educational organisation, faculty and degree course of the student?

**Organisation**	University of Western Sydney
**Faculty**	School of Science and Health
**Degree course**	PhD

2.2.4... Is this research project part of the assessment of the student? Yes

2.2.4... Is the student's involvement in this project elective or compulsory? Compulsory

2.2.4... What training or experience does the student have in the relevant research methodology?

The student has received an Australian Post Graduate Award and UWS top up scholarship to undertake her PhD at UWS. She has been awarded Academic Equivalence for Honours 1 using her her undergraduate track record, publication history and research technician work. She has experience with basic science

research methodology and has used all the techniques described in this application.

2.2.4... What training has the student received in the ethics of research?

As part of her undergraduate degree, the student has completed a number of research subjects as well as an independent research project that required an understanding and knowledge of ethics of research. She has also completed ethics training at the University of Queensland and will undertake more training as part of her PhD at UWS.

2.2.4... Describe the supervision to be provided to the student. NS 4.8.8

Dr Chipchase, Dr Schabrun are the primary co supervisors for this student. Professor Macefield (School of Medicine) is also a co supervisor.

2.2.4... How many supervisors does the student have? 3

2.2.4...Supervisor 1

2.2.4...Provide the name, qualifications, and expertise, relevant to this research, of the students' supervisor

**Title**	Prof
**First Name**	Lucinda
**Surname**	Chipchase
**Summary of qualifications and relevant expertise**	Dr Chipchase has entry level qualifications in physiotherapy, a Masters of Musculoskeletal Physiotherapy and a PhD in physiotherapy. She is experienced in a broad range of research methodologies including randomised controlled trials, clinical trials, observational approaches, including surveys and Delphi questionnaires. In addition, her clinical and theoretical knowledge combined with her hypotheses on their effect at the cortex are central to this application.<br><br>She has expertise in neurophysiological research methods including transcranial magnetic stimulation to probe the motor system; induced pain methodologies and electromyography. She also has expertise in measuring and analysing sensory evoked potentials. Her work in this field to date, has resulted in a number of key publications in neuroscience and physiotherapy journals.<br><br>Due to her varied research experience, she has skills in a variety of quantitative data analysis including parametric and non-parametric statistics.

2.2.4...Supervisor 2

2.2.4...Provide the name, qualifications, and expertise, relevant to this research, of the students' supervisor

**Title**	Dr
**First Name**	Siobhan
**Surname**	Schabrun
**Summary of qualifications and relevant expertise**	Dr Schabrun is an NHMRC post doctoral fellow with a focus on cortical plasticity, non-invasive brain stimulation and neurorehabilitation. She has a doctorate in neurophysiology and an undergraduate honours degree in physiotherapy providing her with strong skills in both basic and clinical research. Her current post-doctoral research (funded by a NHMRC Clinical Research Training Fellowship) extends her work in the field of plasticity into the development and testing of novel rehabilitation strategies. The focus of the current proposal fits directly within this theme.<br><br>Dr Schabrun’s doctoral and post-doctoral work in the field of cortical plasticity have provided her with expertise and strong technical skills in single and paired pulse transcranial magnetic stimulation, electromyography, recording of peripheral Mwaves and use of the Brainsight neuronavigation system. Further she has significant

experience with the recording systems (Signal and Spike software) and analysis methods employed in this proposal. Her expertise in this area is evidenced by 11 full length publications in peer-reviewed international journals (including the prestigious journal Cerebral Cortex, 2 review articles and 1 professional issues paper) that utilize these techniques. This set of skills and expertise is required for successful completion of this project.

In collaboration with Dr Chipchase, Dr Schabrun has authored an invited review and completed two preliminary studies that form the basis for this proposal. The successful completion and publication of this work demonstrates that Dr Schabrun has the skills necessary to ensure the collection of high quality data using the research methods outlined in this proposal.

2.2.4...Supervisor 3

2.2.4...Provide the name, qualifications, and expertise, relevant to this research, of the students' supervisor

**Title**	Prof
**First Name**	Vaughan
**Surname**	Macefield
**Summary of qualifications and relevant expertise**	Vaughan Macefield is Foundation Chair of Integrative Physiology at the School of Medicine, University of Western Sydney, and a Conjoint Senior Principal Research Fellow at Neuroscience Research Australia (NeuRA), formerly Prince of Wales Medical Research Institute. A former NMHRC Senior Research Fellow, he completed his PhD at UNSW in 1986, then undertook advanced training in human neurophysiology in Sweden and the USA. In 1994 he established his own laboratories at NeuRA, prior to joining UWS in 2006.<br>Vaughan specializes in recording from single nerve fibres via tungsten microelectrodes inserted into the peripheral nerves of awake human subjects, and is known internationally as a world expert in recording the firing properties of human sympathetic neurones in health and disease, and as a leading investigator in human sensorimotor control.<br><br>For the last seven years Vaughan has been examining the changes in control of the autonomic nervous system following human spinal cord injury, extending his research into the study of pain and its effects on the autonomic and somatic nervous systems, using brain imaging techniques (fMRI) to study the processing of pain.

2.2.1. Principal researcher / investigator 4

2.2.1. Name and contact details

Name:	Prof Vaughan Macefield
Address:	School of Medicine<br>University of Western Sydney<br>Campbelltown NSW 2751<br>Australia
Organisation:	University of Western Sydney
Area:	School of Medicine
Position:	Professor of Integrative Physiology
Contact	(Bus) 02 4620 3779 (AH) -<br>(Mob) - (Fax) -

Email: v.macefield@uws.edu.au

2.2.2... Summary of qualifications and relevant expertise NS 4.8.7 NS 4.8.15

Vaughan Macefield is Foundation Chair of Integrative Physiology at the School of Medicine, University of Western Sydney, and a Conjoint Senior Principal Research Fellow at Neuroscience Research Australia (NeuRA), formerly Prince of Wales Medical Research Institute. A former NMHRC Senior Research Fellow, he completed his PhD at UNSW in 1986, then undertook advanced training in human neurophysiology in Sweden and the USA. In 1994 he established his own laboratories at NeuRA, prior to joining UWS in 2006. Vaughan specializes in recording from single nerve fibres via tungsten microelectrodes inserted into the peripheral nerves of awake human subjects, and is known internationally as a world expert in recording the firing properties of human sympathetic neurones in health and disease, and as a leading investigator in human sensorimotor control.

For the last seven years Vaughan has been examining the changes in control of the autonomic nervous system following human spinal cord injury, extending his research into the study of pain and its effects on the autonomic and somatic nervous systems, using brain imaging techniques (fMRI) to study the processing of pain.

2.2.2... Please declare any general competing interests

Nil

2.2.2... Name the site(s) for which this principal researcher / investigator is responsible.

University of Western Sydney

2.2.3 Describe the role of the principal researcher / investigator in this project.

Contribute to the development and design of experiments, the analysis and write up of publications where these relate to the research conducted by Emma Jones and in collaborative research projects under this program ethics application.

2.2.4 Is the principal researcher / investigator a student? No

2.3. Associate researcher(s) / investigator(s)

2.3.1 How many known associate researchers are there? (You will be asked to give contact details for these associate researchers / investigators at question 2.3.1.1) 0

2.3.2 Do you intend to employ other associate researchers / investigators? Yes

2.4. Contact

Provide the following information for the person making this application to the HREC.

2.4.1. Name and contact details

Name: Prof Lucinda Chipchase

Address: University of Western Sydney
School of Science and Health
Locked Bag 1797
Penrith NSW 2751
Australia

Organisation: University of Western Sydney

Area: School of Science and Health

Position: Professor of Physiotherapy

Contact (Bus) 0412133210 (AH) -
(Mob) 0412133210 (Fax) 02 4620 3710

Email: l.chipchase@uws.edu.au

2.5. Other personnel relevant to the research project

2.5.1 How many known other people will play a specified role in the conduct of this research project? 1

2.5.1... Describe the role, and expertise where relevant (e.g. counsellor), of these other personnel.

Funding applications (ARC) have requested research assistants. Thus, research assistants may be involved in the conduct of this project. Their role will be largely data collection and analysis.

2.5.2 Is it intended that other people, not yet known, will play a specified role in the Yes

conduct of this research project?

2.6. Certification of researchers / investigators

**2.6.1 Are there any relevant certification, accreditation or credentialing requirements relevant to the conduct of this research?**	No

2.7. Training of researchers / investigators

**2.7.1 Do the researchers / investigators or others involved in any aspect of this research project require any additional training in order to undertake this research?**	No

3. RESOURCES

3.1. Project Funding / Support

3.1.1. Indicate how the project will be funded

3.1.1... Type of funding.
[Please note that all fields in any selected funding detail column (with the exception of the code) will need to be completed.]

	External Competitive Grant	Internal Competitive Grant	By Researchers Department or Organisation
Name of Grant / Sponsor	NHMRC/ARC grants	UWS Internal research grant	UWS School and RHD support
Amount of funding	$500,000	$25000	$7000 p/a
Confirmed / Sought	Sought	Sought	Confirmed
Detail in kind support	Subject reimbursement<br>Full Time Research Associate	Subject reimbursement	Equipment<br>Consumables<br>Subject reimbursement
Indicate the extent to which the scope of this HREC application and grant are aligned	Strongly aligned	Strongly aligned	Strongly aligned

3.1.1... How will you manage a funding shortfall (if any)?
Further smaller grant applications will be made during 2013.

3.1.2 Will the project be supported in other ways eg. in-kind support/equipment by an external party eg. sponsor No

3.1.3... Is this a study where capitation payments are to be made, and will participants be made aware of these payments to clinicians or researchers / investigators? NS 3.3.18b
Not applicable

3.2. Duality of Interest

3.2.1 Describe any commercialisation or intellectual property implications of the funding/support arrangement.
There are no commercialization or IP implications of the funding/support arrangement.

3.2.2 Does the funding/support provider(s) have a financial interest in the outcome of the research? No

3.2.3 Does any member of the research team have any affiliation with the provider(s) of funding/support, or a financial interest in the outcome of the research? No

3.2.4 Does any other individual or organisation have an interest in the outcome of this research No

3.2.5 Are there any restrictions on the publication of results from this research? No

4. PRIOR REVIEWS

4.1. Ethical review

4.1.0. Duration and location

4.1.0... In how many Australian sites, or site types, will the research be conducted? 1

4.1.0... In how many overseas sites, or site types, will the research be conducted? 0

Provide the following information for each site or site type (Australian and overseas, if applicable) at which the research is to be conducted

4.1.0...Site / Site Type 1

4.1.0... Site / Site Type Name
University of Western Sydney

4.1.0... Site / Site Type Location
Neuromodulation and Rehabilitation Laboratory (24.4.123)
School of Science and Health
Campbelltown Campus
NSW

4.1.0...Provide the start and finish dates for the whole of the study including data analysis

**Anticipated start date**	01/07/2013
**Anticipated finish date**	31/12/2017
**4.1.0... Are there any time-critical aspects of the research project of which an HREC should be aware?**	No

4.1.1 To how many Australian HRECs (representing site organisations or the researcher's / investigator's organisation) is it intended that this research proposal be submitted? 1

4.1.1...HREC 1

4.1.1... Name of HREC University of Western Sydney Human Research Ethics Committee (EC00314)

4.1.1...Provide the start and finish dates for the research for which this HREC is providing ethical review.

**Anticipated start date or date range**	01/07/2013
**Anticipated finish date or date range**	31/12/2017
**4.1.1... For how many sites at which the research is to be conducted will this HREC provide ethical review?**	1

4.1.1...Site 1

4.1.1... Name of site University of Western Sydney

4.1.1... Which of the researchers / investigators involved in this project will conduct the research at this site?

Principal Researcher(s)	Associate Researcher(s)
Prof Lucinda Chipchase	
Dr Siobhan Schabrun	
Ms Emma Jones	

4.1.2 Have you previously submitted an application, whether in NEAF of otherwise, for ethical review of this research project to any other HRECs? Yes

4.1.2... To how many other HRECs have you submitted a proposal relating to this research project. 1

4.1.2...HREC 1

4.1.2... Name of HREC The University of Queensland Medical Research Ethics Committee (EC00179)

4.1.2... Status of this review Approved

4.1.2... Explain why an application for ethical review was submitted to the HREC/s identified in answer to question 4.1.2.1, eg. it may be for another phase of the research project which has very different

characteristics. Describe the wider project context, where appropriate.

This was submitted to MREC at UQ when Professor Chipchase worked at UQ. She has now moved to set up her own laboratory at UWS and requires Ethics approval at this institution.

Please provide a copy of the approval letter as an attachment to this application.

4.3. Peer review

4.3.1 Has the research proposal, including design, methodology and evaluation undergone, or will it undergo, a peer review process? NS 1.2

Yes

4.3.1... Provide details of the review and the outcome. A copy of the letter / notification, where available, should be attached to this application.

A similar program ethics application was approved at the Medical Research Ethics Committee at the University of Queensland.
Outcomes as a result of this research have resulted in several peer reviewed papers in high quality journals that have undergone extensive peer review including:

- Schabrun SM, Ridding MC, Galea M, Hodges PW, Chipchase LS (2012): Primary sensory and motor cortex excitability are co-modulated in response to peripheral electrical nerve stimulation. PlosOne (In press, accepted October 31st 2012, PONE-D-12-22553). [IF 4.1].

- Schabrun SM, Chipchase LS, Zipf N, Thickbroom G and Hodges PW (2013): Interaction between simultaneously applied neuromodulatory interventions in humans. Brain Stimulation (accepted for publication 11/10/2012). [IF 3.75; 36/192 Clinical Neurology journals]

- Chipchase LS, Schabrun SM, Cohen L, Hodges PW, Ridding MC, Rothwell J, Taylor J and Ziemann U (2012): A checklist for assessing the methodological quality of studies using transcranial magnetic stimulation to study the motor system: an international consensus study. Clinical Neurophysiology 123 (9): 1698-1704. [IF 3.406, 46/192 of clinical neurology journals]

- Schabrun SM, Cannan A, Mullens R, Dunphy M, Pearson T, Lau C and Chipchase LS (2012): The effect of Interactive Neurostimulation therapy on myofascial trigger points associated with mechanical neck pain: A preliminary randomised, sham-controlled trial Journal of Alternative and Complementary Medicine 18 (10): 946-52. [IF 1.58, 10/22 Integrative and Complementary Medicine]

- Chipchase LS, Schabrun SM and Hodges PW (2011): Corticospinal excitability is dependent on the parameters of peripheral electrical stimulation: a preliminary study. Archives of Physical Medicine and Rehabilitation 92 (9): 1423-1430 [IF 2.284, 10/62 Rehabilitation journals]

Ethical Review Section

Summary

Applicant / Principal Researcher(s)

Prof Lucinda Chipchase

Dr Chipchase has entry level qualifications in physiotherapy, a Masters of Musculoskeletal Physiotherapy and a PhD in physiotherapy. She has experience in a broad range of research methodologies including randomised controlled trials, clinical trials, observational approaches including surveys and Delphi questionnaires. In addition, her clinical and theoretical knowledge combined with her hypotheses on the effect of clinical interventions at the cortex are central to this application.

She has expertise in neurophysiological research techniques outlined in the current proposal including transcranial magnetic stimulation to probe the motor system; induced pain methodologies, electromyography and electroencephalography to measure sensory evoked potentials. Her research in this area has resulted in several publications in high impact neuroscience and physiotherapy journals.

Potential conflicts of interest

No competing interests

Dr Siobhan Schabrun

Dr Schabrun is an NHMRC post doctoral fellow with a focus on cortical plasticity, non-invasive brain stimulation and neurorehabilitation. She has a doctorate in neurophysiology and an undergraduate honours degree in physiotherapy providing her with strong skills in both basic and clinical research. Her current post-doctoral research (funded by a NHMRC Clinical Research Training Fellowship) extends her work in the field of plasticity into the development and testing of novel rehabilitation strategies. The focus of the current proposal fits directly within this theme.

Potential conflicts of interest

There are no competing interests.

Ms Emma Jones

Bachelor of Biomedical Science, University of Newcastle
3 years experience as a Research Technician at the CCRE Spine at the University of Queensland using the techniques outlined in this proposal.

Potential conflicts of interest

There are no competing interests.

Prof Vaughan Macefield

Vaughan Macefield is Foundation Chair of Integrative Physiology at the School of Medicine, University of Western Sydney, and a Conjoint Senior Principal Research Fellow at Neuroscience Research Australia (NeuRA), formerly Prince of Wales Medical Research Institute. A former NMHRC Senior Research Fellow, he completed his PhD at UNSW in 1986, then undertook advanced training in human neurophysiology in Sweden and the USA. In 1994 he established his own laboratories at NeuRA, prior to joining UWS in 2006.
Vaughan specializes in recording from single nerve fibres via tungsten microelectrodes inserted into the peripheral nerves of awake human subjects, and is known internationally as a world expert in recording the firing properties of human sympathetic neurones in health and disease, and as a leading investigator in human sensorimotor control.

For the last seven years Vaughan has been examining the changes in control of the autonomic nervous system following human spinal cord injury, extending his research into the study of pain and its effects on the autonomic and somatic nervous systems, using brain imaging techniques (fMRI) to study the processing of pain.

Potential conflicts of interest

Nil

Other Relevant Personnel

Prof Lucinda Chipchase

Dr Chipchase has entry level qualifications in physiotherapy, a Masters of Musculoskeletal Physiotherapy and a PhD in physiotherapy. She is experienced in a broad range of research methodologies including randomised controlled trials, clinical trials, observational approaches, including surveys and Delphi questionnaires. In addition, her clinical

and theoretical knowledge combined with her hypotheses on their effect at the cortex are central to this application.

She has expertise in neurophysiological research methods including transcranial magnetic stimulation to probe the motor system; induced pain methodologies and electromyography. She also has expertise in measuring and analysing sensory evoked potentials. Her work in this field to date, has resulted in a number of key publications in neuroscience and physiotherapy journals.

Due to her varied research experience, she has skills in a variety of quantitative data analysis including parametric and non-parametric statistics.

Dr Siobhan Schabrun

Dr Schabrun is an NHMRC post doctoral fellow with a focus on cortical plasticity, non-invasive brain stimulation and neurorehabilitation. She has a doctorate in neurophysiology and an undergraduate honours degree in physiotherapy providing her with strong skills in both basic and clinical research. Her current post-doctoral research (funded by a NHMRC Clinical Research Training Fellowship) extends her work in the field of plasticity into the development and testing of novel rehabilitation strategies. The focus of the current proposal fits directly within this theme.

Dr Schabrun's doctoral and post-doctoral work in the field of cortical plasticity have provided her with expertise and strong technical skills in single and paired pulse transcranial magnetic stimulation, electromyography, recording of peripheral Mwaves and use of the Brainsight neuronavigation system. Further she has significant experience with the recording systems (Signal and Spike software) and analysis methods employed in this proposal. Her expertise in this area is evidenced by 11 full length publications in peer-reviewed international journals (including the prestigious journal Cerebral Cortex, 2 review articles and 1 professional issues paper) that utilize these techniques. This set of skills and expertise is required for successful completion of this project.

In collaboration with Dr Chipchase, Dr Schabrun has authored an invited review and completed two preliminary studies that form the basis for this proposal. The successful completion and publication of this work demonstrates that Dr Schabrun has the skills necessary to ensure the collection of high quality data using the research methods outlined in this proposal.

Prof Vaughan Macefield

Vaughan Macefield is Foundation Chair of Integrative Physiology at the School of Medicine, University of Western Sydney, and a Conjoint Senior Principal Research Fellow at Neuroscience Research Australia (NeuRA), formerly Prince of Wales Medical Research Institute. A former NMHRC Senior Research Fellow, he completed his PhD at UNSW in 1986, then undertook advanced training in human neurophysiology in Sweden and the USA. In 1994 he established his own laboratories at NeuRA, prior to joining UWS in 2006.
Vaughan specializes in recording from single nerve fibres via tungsten microelectrodes inserted into the peripheral nerves of awake human subjects, and is known internationally as a world expert in recording the firing properties of human sympathetic neurones in health and disease, and as a leading investigator in human sensorimotor control.

For the last seven years Vaughan has been examining the changes in control of the autonomic nervous system following human spinal cord injury, extending his research into the study of pain and its effects on the autonomic and somatic nervous systems, using brain imaging techniques (fMRI) to study the processing of pain.

5. PROJECT

5.1. Type of Research

5.1.1 Tick as many of the following 'types of research' as apply to this project. Your answers will assist HRECs in considering your proposal. A tick in some of these boxes will generate additional questions relevant to your proposal (mainly because the National Statement requires additional ethical matters to be considered), which will appear in Section 9 of NEAF.

This project involves:

[X] Clinical research NS 3.3
[X] None of the above

5.1.2 Does the research involve limited disclosure to participants? NS 2.3 — Yes

5.1.3 Are the applicants asking the HREC / review body to waive the requirement of consent? NS 2.3.5 — No

5.2. Research plan

5.2.1 Describe the theoretical, empirical and/or conceptual basis, and background evidence, for the research proposal, eg. previous studies, anecdotal evidence, review of literature, prior observation, laboratory or animal studies (4000 character limit). NS 1.1

Background

Persistent musculoskeletal pain has been described as one of the most significant health issues in the developed world. This debilitating condition affects 50 to 80% of the adult population and adversely impacts not only physical and psychological well-being but also the quality of life, employment and economic well-being of sufferers.[1] Despite the scale of the problem, current treatments show only small effect sizes, and most pharmaceutical and behavioural therapies target generic symptoms, not underlying mechanisms.[2-4] A greater understanding of the mechanisms driving the development and persistence of musculoskeletal pain is urgently needed if we are to reduce high recurrence rates and lessen the socioeconomic burden amongst sufferers. Here, we seek to 1) determine the brain and spinal cord mechanisms that underpin the development of persistent pain and 2) develop and test novel physiotherapeutic techniques that target these mechanisms to improve pain and function in those with musculoskeletal pain.

Understanding persistent musculoskeletal pain

Persistent musculoskeletal pain is widely accepted as a biopyschosocial condition for diagnosis and treatment. This means that musculoskeletal pain displays physiological, psychological and social elements.[5] Yet, the contribution of physiological mechanisms to the persistence of pain has received little attention, leading experts to conclude that the ‘bio’ has been overlooked.[6] This situation has hampered our understanding of persistent musculoskeletal pain and stalled the development of therapies with appropriate physiological targets. The identification of physiological mechanisms that underpin the transition to persistent pain is an achievable goal that is likely to have a significant impact on clinical outcomes.

Pain is a multidimensional experience shaped by neural processing across multiple brain regions.[7, 8] Promising physiological mechanisms, such as maladaptive organisation of key brain regions and sensitisation of the brain and spinal cord, have been recently recognized to play a role in persistent pain states.[9] Although these mechanisms are yet to be thoroughly investigated, it has been hypothesized that they underpin the persistence of musculoskeletal pain.[10] In persistent pain, the neuromatrix is thought to be overactive, generating pain in response to smaller or inappropriate stimuli.[11] This sensitisation may be the product of maladaptive reorganisation of brain regions, such that less input is required to trigger the pain neuromatrix.[9] Central sensitisation has also been defined as an increased sensitivity of the brain and spinal cord.[12] The pathophysiology includes altered sensory processing in the brain[13] and increased activity of pain pathways.[14, 15] Together these mechanisms provide a plausible substrate for the persistence of pain.

Despite this, few studies have investigated these mechanisms in acute and persistent musculoskeletal pain. Reorganisation of the sensorimotor cortex has been demonstrated to occur in subjects with fibromyalgia[16], complex regional pain syndrome[17] and low back pain.[18] However, it is unknown whether cortical changes occur in other pain states, how the mechanisms interact and importantly, when in the transition to persistent pain they are active and how they relate to the development of persistent symptoms. Thus, the first stage of this project will examine mechanisms of maladaptive brain organisation and central sensitisation in acute and persistent musculoskeletal pain and in individuals who are not experiencing pain.

This approach will allow us to determine whether these mechanisms are actively involved in the development of persistent musculoskeletal pain.

Treating musculoskeletal pain

Despite intensive global research efforts, persistent musculoskeletal pain remains challenging to treat. Pharmacological, surgical, physical and behavioural therapies are often accompanied by adverse effects or poor patient adherence and have at best, small clinical effects.[19, 20] One explanation for limited clinical effects is that few current therapies target the cortical mechanisms underpinning persistent pain.

Based on information gained in aim one, we will utilise novel physiotherapeutic techniques, such as non-invasive brain stimulation and electrical stimulation of nerves in the periphery, to specifically target mechanisms of brain reorganisation and central sensitisation. Our work and that of others, has demonstrated that physiotherapy techniques, such as peripheral electrical stimulation and non-invasive brain stimulation, are able to induce cortical plasticity with concomitant improvements in function.[21-25] However, further research is required to systematically and rigorously evaluate the effect of these therapies (peripheral electrical stimulation and non invasive brain stimulation) on a range of plasticity indices.[26, 27] If successful, these therapies have the potential to be used as a cortical primer whereby they prime the brain prior to other interventions such as manual therapy and exercise. This has significant potential to reduce the socioeconomic burden associated with musculoskeletal pain.

References

1. Fregni F, Freedman S, Pascual-Leone A: Recent advances in the treatment of chronic pain with non-invasive brain stimulation techniques. Lancet Neurology 2007, 6(2):188-191.
2. Nnoaham Kelechi E, Kumbang J: Transcutaneous electrical nerve stimulation (TENS) for chronic pain. In Cochrane Database of Systematic Reviews. John Wiley & Sons, Ltd; 2008.
3. Karjalainen Kaija A, Malmivaara A, van Tulder Maurits W, Roine R, Jauhiainen M, Hurri H, Koes Bart W: Multidisciplinary rehabilitation for fibromyalgia and musculoskeletal pain in working age adults. In Cochrane Database of Systematic Reviews. John Wiley & Sons, Ltd; 1999.
4. Moore RA, Straube S, Wiffen Philip J, Derry S, McQuay Henry J: Pregabalin for acute and chronic pain in adults. In Cochrane Database of Systematic Reviews. John Wiley & Sons, Ltd; 2009.
5. Waddell G: The Back Pain Revolution. Edinburgh: Churchill LIvingstone; 1998.
6. Hancock MJ, Maher CG, Laslett M, Hay E, Koes B: Discussion paper: what happened to the 'bio' in the bio-psycho-social model of low back pain? European Spine Journal 2011, 20(12):2105-2110.
7. Melzack R: Pain and the neuromatrix in the brain. Journal of dental education 2001, 65(12):1378-1382.
8. Tracey I, Mantyh PW: The cerebral signature and its modulation for pain perception. Neuron 2007, 55(3):377-391.
9. Moseley GL, Flor H: Targeting Cortical Representations in the Treatment of Chronic Pain: A Review. Neurorehabilitation and Neural Repair 2012, 26(6):646-652.
10. Apkarian AV, Hashmi JA, Baliki MN: Pain and the brain: Specificity and plasticity of the brain in clinical chronic pain. Pain 2011, 152(3):S49-S64.
11. Seifert F, Maihoefner C: Central mechanisms of experimental and chronic neuropathic pain: Findings from functional imaging studies. Cellular and Molecular Life Sciences 2009, 66(3):375-390.
12. Nijs J, Meeus M, Van Oosterwijck J, Roussel N, De Kooning M, Ickmans K, Matic M: Treatment of central sensitization in patients with 'unexplained' chronic pain: what options do we have? Expert Opinion on Pharmacotherapy 2011, 12(7):1087-1098.
13. Staud R, Craggs JG, Robinson ME, Perlstein WM, Price DD: Brain activity related to temporal summation of C-fiber evoked pain. Pain 2007, 129(1-2):130-142.
14. Meeus M, Nijs J: Central sensitization: a biopsychosocial explanation for chronic widespread pain in patients with fibromyalgia and chronic fatigue syndrome. Clinical Rheumatology 2007, 26(4):465-473.
15. Meeus M, Vervisch S, De Clerck LS, Moorkens G, Hans G, Nijs J: Central Sensitization in Patients with Rheumatoid Arthritis: A Systematic Literature Review. Seminars in Arthritis and Rheumatism 2012, 41(4):556-567.
16. Salerno A, Thomas E, Olive P, Blotman F, Picot MC, Georgesco M: Motor cortical dysfunction disclosed by single and double magnetic stimulation in patients with fibromyalgia. Clinical Neurophysiology 2000, 111(6):994-1001.
17. Maihoefner C, Baron R, DeCol R, Binder A, Birklein F, Deuschl G, Handwerker HO, Schattschneider J: The motor system shows adaptive changes in complex regional pain syndrome. Brain 2007, 130:2671-2687.
18. Tsao H, Galea MP, Hodges PW: Reorganization of the motor cortex is associated with postural control deficits in recurrent low back pain. Brain 2008, 131:2161-2171.
19. Standaert CJ, Friedly J, Erwin MW, Lee MJ, Rechtine G, Henrikson NB, Norvell DC: Comparative Effectiveness of Exercise, Acupuncture, and Spinal Manipulation for Low Back Pain. Spine 2011, 36(21):S120-S130.
20. Airaksinen O, Brox J, Cedraschi C, Hildebrandt J, Klaber-Moffett J, Kovacs F, Mannion A, Reis S, Staal

J, Ursin H et al: European guidelines for the management of chronic nonspecific low back pain. European Spine Journal 2006, 15(Suppl 2):S192-S300.
21. Schabrun SM, Ridding MC, Galea MP, Hodges PW, Chipchase LS: Primary Sensory and Motor Cortex Excitability Are Co-Modulated in Response to Peripheral Electrical Nerve Stimulation. PLoS One 2012, 7(12):e51298.
22. Schabrun SM, Chipchase LS, Zipf N, Thickbroom GW, Hodges PW: Combined transcranial direct current stimulation (tDCS) and neuromuscular electrical stimulation (NMES) has a competitive effect on central excitability. Society for Neuroscience Abstract Viewer and Itinerary Planner 2012, 42.
23. Chipchase LS, Schabrun SM, Hodges PW: Corticospinal Excitability is Dependent on the Parameters of Peripheral Electric Stimulation A Preliminary Study. Arch Phys Med Rehabil 2011.
24. Chipchase LS, Schabrun SM, Hodges PW: Peripheral electrical stimulation to induce cortical plasticity: A systematic review of stimulus parameters. Clinical Neurophysiology 2011, 122(3):456-463.
25. Schabrun SM, Stinear CM, Byblow WD, Ridding MC: Normalizing Motor Cortex Representations in Focal Hand Dystonia. Cerebral Cortex 2009, 19(9):1968-1977.
26. Schabrun S, Ridding M, Chipchase L: Harnessing the brain's potential to change in musculoskeletal physiotherapy: electrical stimulation as an example. Physiotherapy Theory and Practice 2012, under review.
27. Schabrun SM, Chipchase LS: Priming the brain to learn: The future of therapy? Manual Therapy 2012, 17(2):184-186.

5.2.2 State the aims of the research and the research question and/or hypotheses, where appropriate.

The overall aims of this program ethics application are understand the role of the brain and spinal cord in acute and persistent pain and to develop and test novel therapies that target these mechanisms in musculoskeletal pain states.

The specific aims are:

1. To investigate changes in the topography and excitability of the primary motor and sensory cortex, and changes in spinal cord excitability, in persistent musculoskeletal pain states.

2. To investigate changes in the topography and excitability of the primary motor and sensory cortex, and changes in spinal cord excitability, in acute experimental pain states.

3. To investigate whether physiotherapy interventions can reduce the sensitivity of the brain and spinal cord and normalise brain topography in acute and persistent pain.

5.2.3 Has this project been undertaken previously? Yes

5.3. Benefits/Risks

5.3.0 Does the research involve a practice or intervention which is an alternative to a standard practice or intervention? No

5.3.2 What expected benefits (if any) will this research have for the wider community?

Two benefits are expected from this proposal. First, this work will increase our knowledge of the physiological mechanisms that underpin a major health problem. This information is critical if effective treatment strategies are to be developed that improve patient outcomes and allow practitioners to target treatments to the right people at the right time. Second, we will develop and test novel therapies that if efficacious, will reduce the social and economic burden of persistent musculoskeletal pain.

5.3.3 What expected benefits (if any) will this research have for participants? NS 2.1

There is the potential for participants to derive clinical benefit (e.g. improved pain and function) if they participate in the intervention studies that fall under Aim 2.

5.3.4 Are there any risks to participants as a result of participation in this research project? NS 2.1 Yes

5.3.5 Explain how the likely benefit of the research justifies the risks of harm or discomfort to participants. NS 1.6

The risks to participants is minimal. The results of this research will contribute new knowledge to this field and potentially identify new non-invasive therapies that are effective against chronic pain - a major health problem. This in turn will translate in to a reduction in the socioeconomic costs associated with persistent pain.

5.3.8 Are there any other risks involved in this research? eg. to the research team, the organisation, others Yes

5.3.8... What are these risks?

Research staff involved in inducing experimental pain are at risk of needle stick injuries.

Risks for participants with various measurements are described as follows:

Electromyography (EMG): There are no risks associated with surface EMG where electrodes are placed on the skin after cleaning.

Electroencephalography (EEG): There are no risks associated with EEG, which utilises surface electrodes placed over the scalp to measure brain activity.

TMS: Transcranial magnetic stimulation (TMS) is a widely utilized safe non-invasive technique for the investigation of the motor cortex in human individual. We have significant experience with this technique. One risk that has been suggested in the literature is the accidental induction of a seizure. However, in the last 20 years of TMS usage, there have been no reported cases of accidental induction of seizures using single or paired pulse TMS in healthy individuals with no cortical lesions or abnormalities. Nevertheless, seizures have been produced in several patients with large cerebral infarcts or other structural lesions.1-3 There is also a small risk (0.0-3.6 percent) of inducing a seizure in people with epilepsy. 4

The inclusion of the latest safety guidelines5 to inform the participants of this risk and to exclude subjects with brain lesions or a history of epilepsy should prevent adverse incidence from occurring. In the unlikely event of an accidental first time seizure in a subject, standard UWS emergency procedure would be implemented. All research investigators and assistants will be well-versed in these standard emergency procedures. Consistent with this, appropriate first aid would be provided, an ambulance called and the subjects taken to hospital. As the risk of seizure is extremely low and proposed emergency procedures consistent with standard practices for first ever seizures, it is not necessary for a medical practitioner to be present for testing.

Transcranial direct current stimulation: tDCS is a non-invasive, painless and safe technique which is used worldwide. The use of tDCS in healthy subjects and across a range of pathological conditions has not resulted in any significant adverse effects8. tDCS produces a mild tingling or itching sensation over the scalp when the machine is first turned on. This sensation subsides within the first couple of minutes. Mild symptoms of fatigue (35 %), mild discomfort (15 %), headache (5 %) and nausea lasting not more than 2 hours (3 %) have been reported following tDCS. 6 Subjects will be fully briefed on each of these symptoms and be given the opportunity to withdraw at any time. The presence of these symptoms will be monitored verbally by the investigator and tDCS terminated if the subject becomes uncomfortable. Subjects who have a history of frequent or severe headaches/migraines will be excluded from participation.

Experimental pain: The injection of hypertonic saline induces short term pain that can be carefully controlled. Pain resembles normal muscle pain and is of moderate intensity. The painful sensation lasts only a short time with complete recovery within 20-30 minutes. This procedure has been used extensively in our prior work. Risks are minimal and are associated with feelings of dizziness or light headedness. This is dealt with proactively and preventative steps will be taken. These include having the subject lie down when any symptom of sweating, light headed sensation or dizziness is reported.

Electrical stimulation: Electrical stimulation is a common non-invasive treatment used by physiotherapists and other health care providers as well as being available to the public 'over the counter'. The main risk from electrical stimulation applied through the skin is to the skin itself. This risk is very small with skin irritation a very rare side effect. Electrical stimulation can be applied for a number of hours with little risk.7 As the electrical stimulation will be applied for no more than 1 hour, the risk of skin irritation is high unlikely. However, to limit this risk even further, electrodes will be replaced regularly and the skin checked before and after treatment. In the unlikely event of skin irritation, subjects will be instructed to see their local general practitioner. All electrical stimulation application will follow the Australian Physiotherapy Association Guidelines for use of Electrophysical Agents.8

Pressure and thermal pain thresholds: There are no risks associated with measuring pressure or thermal pain thresholds. Some discomfort may be present when pressure/thermal sensations turn to pain. However, this feeling is transient and is ceased as soon as subjects indicate the sensation has changed.

References

1. Fauth C, Meyer BU, Prosiegel M, et al. Seizure induction and magnetic brain stimulation after stroke. Lancet 1992;339:8789.
2. Hallett M. Transcranial magnetic stimulation and the human brain. Nature 2000;406:147-150.
3. Kandler R. Safety of transcranial magnetic stimulation. Lancet 1990;335:469-70.
4. Schrader LM, Stern JM, Koski L, et al. Seizure incidence during single- and paired-pulse transcranial magnetic stimulation (TMS) in individuals with epilepsy. Clinical Neurophysiology 2004;115:2728-2737.
5. Wassermann EM. Risk and safety of repetitive transcranial magnetic stimulation: report and suggested guidelines from the International Workshop on the safety of repetitive transcranial magnetic stimulation

1996. Electroencephalography and Clinical Neurophysiology 1998;108:1-16.
6. Poreisz C, Boros K, Antal A, et al. Safety aspects of transcranial direct current stimulation concerning healthy subjects and patients. Brain Research Bulletin 2007;72:208-214.
7. Robertson V WA, Low J, Reed A Electrotherapy Explained: Principles and Practice Edinburgh: Butterworth Heinemann. 2006.
8. Robertson VJ, Chipchase L, Laakso E, Whelan K and McKenna L (2001) Guidelines for the clinical use of electrophysical agents. Australian Physiotherapy Association 2001.

5.3.8... Explain how these risks will be negated/minimised/managed.

Needle stick injuries: All staff will be trained on the use of personal protective equipment and appropriate use and disposal of needles (i.e. correct usage of sharps removal containers). Personal protective equipment will be enforced when handling human blood and needles.

How the risks to participants will be minimised is outlined in the previous sections along with references to support the level of risk and intended strategies.

5.3.8... Explain how these risks will be monitored.

All four participants have experience from prior work in the induction of acute pain through saline injections. Dr Chipchase and Dr Schabrun will ensure ongoing competence of the current investigators and will train any new researchers that join this program of research.

5.3.8... Explain how any harm to participants, resulting from these risks, will be reported.

Any incidents will be immediately reported to the Dean (School of Science and Health) and the Work Health and Safety Coordinator. A full incident report and risk assessment will be lodged within 48 hours. In addition, any adverse events will also be reported to the Human Ethics Officer.

5.3.9 Is it anticipated that the research will lead to commercial benefit for the investigator(s) and or the research sponsor(s)? No

5.3.11 Is there a risk that the dissemination of results could cause harm of any kind to individual participants - whether their physical, psychological, spiritual, emotional, social or financial well-being, or to their employability or professional relationships - or to their communities? No

5.4. Monitoring

Refer to NS 3.3.19 - 3.3.25

5.4.1 What mechanisms do the researchers / investigators intend to implement to monitor the conduct and progress of the research project? NS 5.5

Two researchers will always be on site during the data collection.

The rules and regulations of the UWS laboratory require regular progress report updates. Regular meetings between researchers will be scheduled and progress reports/updates will be provided to the principle investigator.

5.4.2 Please detail your Data and Safety Monitoring Board (DSMB) and its nominee for this trial. NS 3.3.20(c)

N/A

6. PARTICIPANTS

6.1. Research participants

6.1.1 The National Statement identifies the need to pay additional attention to ethical issues associated with research involving certain specific populations.

This question aims to assist you and the HREC to identify and address ethical issues that are likely to arise in your research, if its design will include one or more of these populations. Further, the National Statement recognizes the cultural diversity of Australia's population and the importance of respect for that diversity in the recruitment and involvement of participants. Your answer to this question will guide you to additional questions (if any) relevant to the participants in your study.

6.1.1 Tick as many of the following 'types of research participants' who will be included because of the project design, or their inclusion is probable, given the diversity of Australia's population. If none apply, please indicate this below.

	c) Design specifically excludes
Women who are pregnant and the human foetus NS 4.1	[X]

6.2. Participant description

6.2.1 How many participant groups are involved in this research project? 2

6.2.2 What is the expected total number of participants in this project at all sites?
Approximately 200 individuals will be recruited in equal male and female ratios. Final numbers for each experiment will be based on appropriate power calculations.

6.2.3. Group 1

6.2.3... Group name for participants in this group
Adults with musculoskeletal or induced muscle pain

6.2.3... Expected number of participants in this group
100

6.2.3... Age range
18-65

6.2.3... Other relevant characteristics of this participant group
Participants in this group will either include;

1. Healthy indivudials who will recieve an injection of hypertonic saline to inducte acute muscle pain.

2. Participants will be experiencing persistent musculoskeletal pain (e.g. low back or arm pain). Participants must have experienced symptoms for at least 3 months, which have been severe enough for participants to have sought treatment from a health professional. Symptoms must be current at the time of testing. Subjects will be excluded is they are less than 18 years old, have other major diseases/disorders (e.g. chronic renal/endocrine disorders), psychiatric or neurological conditions, are cognitively impaired, are pregnant, have epilepsy or metal implants (such as plates and/or screws) in the head and neck.

6.2.3... Why are these characteristics relevant to the aims of the project?
Given the aims of the research and due to the heterogeneity of musculoskeletal pain it is imperative that potential confounding variables are controlled. These criteria aim to minimise the impact of major potential confounders and ensure the most representative participants are included.

6.2.3. Group 2

6.2.3... Group name for participants in this group
Healthy subjects

6.2.3... Expected number of participants in this group
100

6.2.3... Age range
18-65

6.2.3... Other relevant characteristics of this participant group
Participants who have no history of musculoskeletal pain in the back or arm will be included. These participants will be either i) age and gender matched to individuals recruited in Group 1 or ii) included in experiments of the effect of acute pain (induced via hypertonic saline injection) on the brain and spinal cord.

6.2.3... Why are these characteristics relevant to the aims of the project?

Given the aims of the research it is important that i) participants in this group are age and gender matched to participants with musculoskeletal pain in order to accurately compare data between groups or ii) participants are pain free to allow the effects of acute pain to be accurately determined.

6.2.4. Your response to questions at Section 6.1 - Research Participants indicates that the following participant groups are excluded from your research. If this is not correct please return to section 6.1 to amend your answer.

Women who are pregnant and the human foetus

6.2.4... Have any particular potential participants or groups of participants been excluded from this research? In answering this question you need to consider if it would be unjust to exclude these potential participants. NS 1.4

Individuals will be excluded if they have any of the following: medically diagnosed or suspected epilepsy, brain lesions or infarcts, circulatory/neurological/respiratory lesions or disorders, are cognitively impaiared, recent pregnancies or currently pregnant, or any implanted orthopaedic or metallic items.

These exclusions are based on safety guidelines for working with TMS. Participants with an intellectual or mental impairment are also excluded as the study involves detailed movement examination and questionnaires that require comprehension of instructions. The design and methodology is not appropriate or applicable to children and/or young people with or without persistent pain. People highly dependent on medical care represent a subgroup with needs that are not relevant to the purpose of this study.

6.3. Participation experience

6.3.1 Provide a concise detailed description, in not more than 200 words, in terms which are easily understood by the lay reader of what the participation will involve.

Participation in this study may involve one or more of the following procedures.

a) To measure activity of your limb muscles, sensors will be placed on the skin overlying the muscles. The specific muscles to be recorded will be explained to you prior to the study.

b) To evaluate the function of the nerve to the muscle the response of the muscle to a brief electrical stimulus will be assessed. A small brief current will be applied to the nerve via electrodes placed on the skin and the intensity will be adjusted to evaluate the size of the response. This procedure is associated with a sensation of mild pins and needles and is similar to some electrotherapy applications used by physiotherapists.

c) A brief train of electrical stimulation will be applied to your limb muscles. The intensity of the stimulation will be adjusted until we observe a small reflex. You will be asked to tell the investigator when the sensation changes from pins and needles to pain.

d) Transcranial magnetic stimulation (TMS) will be used to evaluate the activity of cells of your brain that control your muscle. The technique involves a brief magnetic field that induces changes in the electrical activity of the brain directly under the stimulator. The stimulator is placed over the region of the brain responsible for control of movement. When stimulated, an electrical signal is delivered to the muscle to make it contract. We detect this muscle activity via sensors placed on the skin. You will be asked to wear a pair of glasses to ensure accurate location of the magnetic stimulation. A loud click accompanies the stimulus and sound and feels like flicking a bike helmet while it is on your head. You will receive either one or two TMS pulses close together but at varying intervals.

e) To evaluate the effect of experimental pain on your muscles, a hypertonic saline solution (salty water) will be injected. This will be done as either a single injection or as an initial injection followed by a continuous infusion into your muscle. Hypertonic saline is water that has a higher salt concentration than your body tissues and injection of it into your muscle will produce short-term pain that we can carefully control. Pain resembles normal muscle pain and is of moderate intensity. The painful sensation from an injection lasts only a short time with complete recovery within 20-30 minutes. A continuous infusion may be used to allow the painful session to last slightly longer up to 45 minutes.

f) Transcranial direct current stimulation (tDCS): tDCS is a non-invasive, painless and safe technique which is used worldwide. tDCS produces a mild tingling or itching sensation over the scalp when the machine is first turned on. This sensation subsides within the first couple of minutes.

g) Peripheral electrical stimulation: This is a common physiotherapy intervention. An electrical stimulation device will be applied to your body using electrodes. You will feel a tingling sensation under the electrodes and you may also feel the muscles contract similar to if you did it yourself. The device will be applied to your skin using re-usable electrodes. These devices are used commonly in physiotherapy practice and provide

very few risks.

Depending on the specific study and the methods included above, the time commitment for participants will vary. Testing sessions are likely to be between 1-3 hours and there may be a requirement for repeat testing. Testing will take place in the Neuromodulation and Rehabilitation Laboratory that is located in Building 24 (42..3.123) on the Campbelltown Campus.

6.4. Relationship of researchers / investigators to participants

6.4.1 Specify the nature of any existing relationship or one likely to rise during the research, between the potential participants and any member of the research team or an organisation involved in the research.

There is likely to be no relationship between the participants and investigators. However, there is a small chance that a staff member from UWS offers to be participants.

6.4.2 Describe what steps, if any, will be taken to ensure that the relationship does not impair participants' free and voluntary consent and participation in the project.

Participants will be informed that they are free to withdraw from the study at any time, without stating a reason, without affecting their relationship with the researchers.

6.4.3 Describe what steps, if any, will be taken to ensure that decisions about participation in the research do not impair any existing or foreseeable future relationship between participants and researcher / investigator or organisations.

Participants will be informed of their right to withdraw from the study at any time without prejudice and that this will not impact on their relationship with the organisation or researchers.

Any decision made in relation to the research project will not impact on any future relationship with researchers or investigators.

6.4.4 Will the research impact upon, or change, an existing relationship between participants and researcher / investigator or organisations.? No

6.5. Recruitment

6.5.1 What processes will be used to identify potential participants?

Posters, internet databases, social media, advertisements in University News and local newspapers, medical and physiotherapy clinics and sporting clubs will be used to identify potential participants. Participants will be targeted within Campbelltown and surrounding areas for ease of attendance.

6.5.2 Is it proposed to 'screen' or assess the suitability of the potential participants for the study? Yes

6.5.2... How will this be done?

An information sheet and consent form detailing the aims, procedures, risks and benefits as well as the inclusion and exclusion criteria will be given to each subject to determine their eligibility and willingness to participate in this study. Screening will be conducted by the study team through email, phone, or onsite at UWS. Subjects will also be required to complete a medical history questionnaire and a safety-screening questionnaire to the use of transcranial magnetic stimulation prior to participation.

6.5.3 Describe how initial contact will be made with potential participants.

Once subjects have been identified and have provided contact details to the investigators, they will then be initially contacted via telephone call and/or email correspondence. During this communication the researcher will review the study inclusion/exclusion screening questionnaire to deem eligibility, and if appropriate, schedule the research appointment. If the participant is deemed ineligible, the reason for not including them in the study will be explained to them.

6.5.3... Do you intend to include both males and females in this study? Yes

6.5.3... What is the expected ratio of males to females that will be recruited into this study and does this ratio accurately reflect the distribution of the disease, issue or condition within the general community?

We expect an equal ratio of males and females will be recruited. 50:50

6.5.4 Is an advertisement, e-mail, website, letter or telephone call proposed as the form of initial contact with potential participants? Yes

6.5.4... Provide details and a copy of text/script.

Volunteer Opportunity

People aged between 18 and 65 who have pain in one or both arms are needed for a research study into the brain mechanisms behind persistent muscle pain. By conducting this study we hope to find out why some people get better after injury while others go on to develop a persistent or reoccurring condition. All procedures are non-invasive and conducted at University of Western Sydney, Campbelltown campus. All volunteers must have experienced their pain for at least 3 months to be eligible. All volunteers will be

reimbursed for their travel and parking expenses. For more information please call or email X_______________.

6.5.5 If it became known that a person was recruited to, participated in, or was excluded from the research, would that knowledge expose the person to any disadvantage or risk? No

6.6. Consent process

6.6.1 Will consent for participation in this research be sought from all participants? Yes

6.6.1... Will there be participants who have capacity to give consent for themselves? Yes

6.6.1... What mechanisms/assessments/tools are to be used, if any, to determine each of these participant's capacity to decide whether or not to participate?

The study excludes those who are not eligible to give consent for themselves based on age and cognitive capacity. The screening process that includes a patient interview will ensure those individuals with insufficient cognitive capacity will be identified on the basis of their ability to complete study documentation.

6.6.1... Are any of the participants children or young people? No

6.6.1... Will there be participants who do not have capacity to give consent for themselves? No

6.6.1... Describe the consent process, ie how participants or those deciding for them will be informed about, and choose whether or not to participate in, the project.

Participants will be provided with a subject information statement and consent form. The investigator will also verbally outline the procedures to be used in the study and any risks involved. Any questions related to these forms will be answered. The information sheet and consent form will clearly outline the intended use of data collected from the participant. This will include data analyses for the current project and potential for subsequent analyses in related studies. After confirmation of their suitability for the trial the participant will be asked to sign the consent form. Consent forms will be signed prior to commencement of the study.

6.6.1... If a participant or person on behalf of a participant chooses not to participate, are there specific consequences of which they should be made aware, prior to making this decision? 4.6.6 - 4.6.7

No

6.6.1... Might individual participants be identifiable by other members of their group, and if so could this identification expose them to risks?

No

6.6.1... If a participant or person on behalf of a participant chooses to withdraw from the research, are there specific consequences of which they should be made aware, prior to giving consent?

No

6.6.1... Specify the nature and value of any proposed incentive/payment (eg. movie tickets, food vouchers) or reimbursement (eg travel expenses) to participants.

Participants will be reimbursed a nominal amount to cover the cost of travel/parking to the testing sessions. In previous laboratories in which I have worked, the nominal amount has been $15 per hour and this will be offered at UWS.

6.6.1... Explain why this offer will not impair the voluntary nature of the consent, whether by participants' or persons deciding for their behalf. NS 2.2.10 - 2.2.11

The payment is minimal and is only intended to cover travel expenses and fees associated with parking at the venue. Participants will be required to travel to the testing laboratory for a single session that may last approximately 3 hours.

6.6.3 Do you propose to obtain consent from individual participants for your use of their stored data/samples for this research project? No

6.6.3... Give justification

Data files will only be accessed for further analyses in an exploratory manner.

8. CONFIDENTIALITY/PRIVACY

8.1. Do privacy guidelines need to be applied in the ethical review of this proposal?

8.1.1 Indicate whether the source of the information about participants which will be used in this research project will involve:

[X] collection directly from the participant

8.1.1... Information which will be collected for this research project directly from the participant

8.1.1... Describe the information that will be collected directly from participants. Be specific where appropriate.

Transcranial magnetic stimulation (TMS) can measure a number of indices of cortical plasticity including excitability, organisation and inhibition.

Excitability
Cortical excitability of the motor system will be investigated by applying a single TMS pulse to the scalp and measuring the response. The response, known as a motor evoked potential (MEP) will be measured by EMG electrodes over the muscle of interest.

Organisation
The cortical representation of a muscle is estimated by applying a single TMS pulse to each point on a grid defined by a neuro-navigation system and measuring the average response at each point. The average amplitude of the MEP is then graphically presented to produce a map of the muscles representation. Comparisons can then be made between the size and location of the cortical representation for each muscle in those with chronic pain and healthy controls. The reliability of this technique has been previously demonstrated (Uy et al., 2002). Three variables from the motor topography will be calculated and used for analysis: centre of gravity, map area and maximum MEP.

Inhibition
Corticocortical circuitry will be investigated using a measure of inhibition known as short-interval intracortical inhibition (SICI). TMS will be applied with two pulses. The first conditioning stimulus from the TMS will be sub-threshold and set at an intensity of 5% below the threshold for evoking responses in the tonically active target muscle. The second stimulus will be at a supra threshold level to evoke an MEP of approximately 1 mv in the relaxed target muscle. Three different interstimulus intervals (3ms, 5ms and 13 ms) will be tested randomly to provide a time course of the inhibitory effects.

Electroencephalographic (EEG) recordings
Electrical stimulation of sensory nerves will be used to measure the responsiveness of the sensory cortex. This is achieved by giving a small non-painful electrical stimulus to the nerve at the wrist and measuring the response in the cortex. Surface electrodes will be placed over the scalp to record cortical responses.

Electromyographic (EMG) recordings
Surface electromyography (EMG) electrodes will be used to to record muscle activity. EMG data will be taken from trunk and/or limb muscles using electromyography equipment. Standard silver/silver chloride electrodes placed on the skin which allows us to pick up the signal in the muscle that has been evoked by transcranial magnetic stimulation (TMS).

Peripheral electrical stimulation
Peripheral electrical stimulation (PES) will be used in this study in several ways.
1.The response of the muscle to direct stimulation of the nerve will be used to examine the responses evoked by TMS.
2.Electrical stimulation of sensory nerves will be used to measure the responsiveness of the sensory cortex. This is achieved by giving a small non-painful electrical stimulus to the nerve at the wrist and measuring the response in the cortex.
3.A train of electrical stimulation will be given to the nerves at the ankle to assess the sensitivity of spinal pathways in healthy individuals and in those with pain.
4.Electrical stimulation, identical to that used in physiotherapy practice, will be be used as an intevention.

Experimental Pain
The effect of experimentally induced pain on excitability and organisation of the motor cortex will be assessed by injection of hypertonic saline. Hypertonic saline solution (3-8% saline) will be injected either as a single bolus injection (0.1-5ml) or as a bolus injection followed by a continuous infusion (1-10ml/hour via a syringe driver) into muscle tissue or other soft tissue. The injection of hypertonic saline induces short term

pain that can be carefully controlled. Pain resembles normal muscle pain and is of moderate intensity. The painful sensation lasts only a short time with complete recovery within 20-30 minutes.

Pressure and thermal pain thresholds
Pressure pain thresholds (PPTs) will be measured using a pressure algometer. Subjects will be requested to push a button when the sensation changes from one of pressure to pain. Thermal pain thresholds will be measured using the Thermotest system. Subjects will be asked to press a button when the sensation first becomes painful.

Questionnaires
Participants will be asked to fill out a general screening tool which includes contraindications to various methods as well as demographic information such as age and gender.

In addition, participants may be required to complete some standard questionnaires in which they are asked to report their pain, function and psychosocial wellbeing. For example, subjects maybe required to provide answers to the following questionnaires

1.Pain Catastrophising scale (PCS): 14 Likert items (04) that assess coping strategies and catastrophic thoughts about pain. It has strong construct validity, reliability and stability. There are three sub-factors of the PCS: worry, passive coping and helplessness.

2. Centre for Epidemiologic Studies Depression Scale (CESD): 20 items, measuring feelings over past week; 6 subscales, total between 0–60, and a cut point of 16.
3. Fear Avoidance Beliefs Questionnaire (FABQ):16 items, 5 related to physical harm, and 11 related to fears about work; responses on a 0–6 Likert scale.

4. General pain in the last week: 11 point Numerical Rating Scale.

5. Roland Morris Disability Questionnaire (RMDQ): 28 yes/no responses to what you can/ can't do because of your persistent pain.

6. Demographic information including age, gender, handedness.

8.1.1... The information collected by the research team about participants will be in the following form(s). Tick more than one box if applicable.
[X] individually identifiable

8.1.1... Give reasons why it is necessary to collect information in individually identifiable or re-identifiable form.
Data collection will include the participants name, contact details (email and phone) and demographic details such as age and gender. This is necessary so we can screen the subject for age (> 18 only) and also contact the subject if further testing is required.

The participants name will only be included on the screening form. On this form, the participant will be assigned a number so that all future data collection will be in a de-identified form.

8.1.1... Consent process

You have indicated that you will be varying the conditions of or waiving consent. See questions in section 6.6

8.1.1... Will consent be specific or extended or unspecified? NS 2.2.14 - 2.2.18 Extended

8.1.1... Provide reasons why this form of consent has been chosen. You may need to revise your answer at 6.6.1.1.3 to provide details on the consent process
While this is a program ethics application, the information sheet includes all forms of potential data collection. Only those that will be used in that specific experiment will be ticked and discussed with the participant. Participants will consent based on this specific methods and further approval of the specific information sheet from the HREC. If participants would like to be involved in future but similar projects, that fall within this program ethics, then consent will be gained again.

8.2. Using information from participants

8.2.1 Describe how information collected about participants will be used in this project.
Information collected will be used for analysis purposes only, and will be reported as group data, no individual participant will be identified.

8.2.2 Will any of the information used by the research team be in identified or re-identifiable (coded) form? Yes

8.2.2... Indicate whichever of the following applies to this project:

[X] Information collected for, used in, or generated by, this project will not be used for any other purpose.

8.2.4 List ALL research personnel and others who, for the purposes of this research, will have authority to use or have access to the information and describe the nature of the use or access. Examples of others are: student supervisors, research monitors, pharmaceutical company monitors .

Professor Lucy Chipchase will maintain the screening forms and any data files with personal information in a locked filing cabinet in her office. Dr Chipchase and Schabrun will have access to all data files for data analysis.

Ms Emma Jones will have access to relevant de-identified electronic files related to her Phd studies and any other study in which she contributes.

Professor Macefield will have access to relevant electronic de-identified data files for any particular project with which he is involved.

If further research higher degree students, research assistants or post doctoral researchers join the laboratory and complete research that falls under this program ethics, then they will have access to relevant data files.

Research monitors and pharmaceutical companies will not have access to any data files.

8.3. Storage of information about participants during and after completion of the project

8.3.1 In what formats will the information be stored during and after the research project? (eg. paper copy, computer file on floppy disk or CD, audio tape, videotape, film)

Data will be stored both in paper copy and on computer file. Each participant will be allocated an identification code in order to protect his or her identity. Any data collected, will then be recorded with respect to their identification code. Personal information collected will be stored in a secure database with access only by investigators involved in the research. All information obtained from this experiment will be treated confidentially. Publications resulting from this study will reveal the information in a manner that does not identify the subjects involved. Subjects will be informed of the potential risks and confidentiality issues in the information sheet. At all times, the confidentiality of subjects, the personal information and the recorded data will be maintained at the standards in accordance to the National Health and Medical Research Council's guidelines.

8.3.2 Specify the measures to be taken to ensure the security of information from misuse, loss, or unauthorised access while stored during and after the research project? (eg. will identifiers be removed and at what stage? Will the information be physically stored in a locked cabinet?)

All hardcopies of data will be kept in a locked filing cabinet in the office (Campbelltown Campus, Building 24, level 4, room 115) of the chief investigator for the duration of the study when not in use. Computer data will be de-identified and stored in a personal drive of a university computer, with a backup burnt to CD that will be kept with the hard copy data in a locked cabinet.

8.3.5 The information which will be stored at the completion of this project is of the following type(s). Tick more than one box if applicable.

[X] non-identifiable

8.3.6 For how long will the information be stored after the completion of the project and why has this period been chosen?

Data will be destroyed after 5 years from the publication of the data based on minimum NHMRC requirements (Joint NHMRC/AVCC Statement 1997).

8.3.7 What arrangements are in place with regard to the storage of the information collected for, used in, or generated by this project in the event that the principal researcher / investigator ceases to be engaged at the current organisation?

Arrangements will be made for the information to be stored in a secure filing cabinet within the School of Science and Health (Campbelltown 24.4.15).

8.4. Ownership of the information collected during the research project and resulting from the research project

8.4.2 Who is understood to own the information resulting from the research, eg. the final report or published form of the results?

The data is owned by the investigators and The University of Western Sydney (this is in line with current conditions of employment and study at The University of Western Sydney). The copyright of any published papers will be assigned to the publisher of the journal (as is current practice).

8.4.3 Does the owner of the information or any other party have any right to impose limitations or conditions on the publication of the results of this project? No

8.5. Disposal of the information

8.5.1 Will the information collected for, used in, or generated by this project be disposed of at some stage? Yes

8.5.1... At what stage will the information be disposed?
5 years from study completion.

8.5.1... How will information, in all forms, be disposed?
Paper will be shredded and CD/DVDs, data files and paper will be disposed of in locked waste disposal bins for confidential disposal.

8.6. Reporting individual results to participants and others

8.6.1 Is it intended that results of the research that relate to a specific participant be reported to that participant? No

8.6.1... Explain/justify why results will not be reported to participants.
There is no intention to share the results of a specific participant to that participant as raw data requires a significant amount of analysis prior to understanding the potential outcome. However, if requested, we will inform the participant of his/her scoring on the questionnaires and laboratory measures after completion of the research project.

8.6.2 Is the research likely to produce information of personal significance to individual participants? No

8.6.3 Will individual participant's results be recorded with their personal records? No

8.6.4 Is it intended that results that relate to a specific participant be reported to anyone other than that participant? No

8.6.5 Is the research likely to reveal a significant risk to the health or well being of persons other than the participant, eg family members, colleagues No

8.6.6 Is there a risk that the dissemination of results could cause harm of any kind to individual participants - whether their physical, psychological, spiritual, emotional, social or financial well-being, or to their employability or professional relationships - or to their communities? No

8.6.7 How is it intended to disseminate the results of the research? eg report, publication, thesis
Thesis.
Conference and peer reviewed journal publications.

8.6.8 Will the confidentiality of participants and their data be protected in the dissemination of research results? Yes

8.6.8... Explain how confidentiality of participants and their data will be protected in the dissemination of research results
Dissemination of results will involve only group data. Individual data will data will not be used in publications and will remain de-identified.

9. PROJECT SPECIFIC

9.1. Type of research/trial

**9.1.1 The administration of a drug / medicine (includes a complementary / alternative medicine)**	No
**9.1.2 The use of a medical device**	Yes
**9.1.3 The administration of human somatic cell gene therapy**	No
**9.1.4 The use of a xenotransplant**	No
**9.1.5 The use of stem cells (adult or embryonic) as therapy**	No
**9.1.6 Other**	No

9.1.7. The project will be conducted as follows:

**9.1.7... Under the Clinical Trial Notification Scheme (CTN)**	No
**9.1.7... Under the Clinical Trial Exemption Scheme (CTX)**	No

You have indicated that you are conducting a clinical trial under neither the CTN or CTX scheme. Please ensure that this is correct by referring back to your answer at Q 5.1.1.2. If you are conducting a trial in a clinical setting, which will not take place under CTN or CTX, please ensure that enough detail has been provided about the research to allow a HREC to adequately review it. This may require you to review your answers in section 5.1.1 and/or 6.1.1.

9.1.8. Provide the following details for the clinical trial protocol:

9.1.8... Protocol name
This is not a clinical trial protocol

9.1.8... Protocol version number
N/A

9.1.8... Protocol version date 20/04/2013

9.1.8... If you intend to/have registered this trial in a publicly accessible register, please provide the details of it here NS 3.3.12
N/A

9.1.9. Provide the following details for the investigator's brochure/product information (as relevant):

9.1.9... Title of Investigator's Brochure
N/A

9.1.9... Investigator's brochure version number
N/A

9.1.9... Investigator's brochure version date 20/04/2013

9.1.1... Use of a medical device under the CTN/CTX scheme

9.1.1... Describe the trial phase for the medical device.
This is not a clinical trial but medical devices such as transcutaneous electrical stimulation will be tested.

9.1.1... How many devices, including comparators, are being tested in the trial? 1

9.1.1... Provide the following information for each device

9.1.1...Device 1

9.1.1... Approved name
NA

9.1.1... Trade name
NA

9.1.1... Is the device implantable? No

9.1.1... Known adverse effects
NA

9.1.1... Known contra-indications/warnings

NA

9.1.1... Length of time participants will be monitored for adverse reactions
NA

9.1.1... Has the sponsor or manufacturer agreed to supply the device free of charge for the duration of the trial? No

9.1.1... Describe what arrangements have been made for the supply of the device.
NA

9.1.1... Non-implantable Device

9.1.1... Will the trial device/treatment be made available to participants after the completion of the trial? No

9.1.1... Explain why participants will not have post trial access to the device.
NA

9.2. Clinical research

9.2.1. The study examines:

9.2.1... The administration of a drug / medicine (includes a complementary / alternative medicine) No

9.2.1... The use of a medical device No

9.2.1... Other No

9.2.2. Provide the following details for the study protocol:

**Protocol title**	NA
**Protocol version number**	NA
**Protocol version date**	01/01/2014

9.2.3. Provide a statement describing the following:

9.2.3... Method of randomisation
NA

9.2.3... Whether the hypothesis offers a realistic possibility that the intervention is at least as effective as standard treatment.
NA

9.2.3... The justification for the use of placebo or non-treatment control group, including alternative effective treatments and any risk of harm in the absence of treatment.
NA

9.2.3... How variations in response will be treated
NA

9.2.3... Endpoints
NA

9.2.3... Details of contingencies and management of these
NA

9.2.3... Explain the arrangements in place to ensure there is adequate compensation for participants.
NA

9.2.4 How many drugs will be used in this research project? 1

9.2.5. Provide the following information for each drug

9.2.5. Drug 1

9.2.5... Approved name
NA

9.2.5... Trade name
NA

9.2.5... Approved therapeutic indication, dose and duration in Australia
NA

9.2.5... Dosage regimen

NA

9.2.5... Known adverse effects

NA

9.2.5... Known contra-indications/warnings

NA

9.2.5... Concurrent drugs to be avoided

NA

10. DECLARATIONS AND SIGNATURES

10.1 Project Title

Pain, physiotherapy and underlying cortical mechanisms

10.2 Human Research Ethics Committee to which this application is made

The University of Queensland Medical Research Ethics Committee (EC00179)

University of Western Sydney Human Research Ethics Committee (EC00314)

10.3 Signatures and undertakings

Applicant / Principal Researchers (including students where permitted)

I/we certify that:

- All information is truthful and as complete as possible.
- I/we have had access to and read the National Statement on Ethical Conduct in Research Involving Humans.
- the research will be conducted in accordance with the National Statement.
- the research will be conducted in accordance with the ethical and research arrangements of the organisations involved.
- I/we have consulted any relevant legislation and regulations, and the research will be conducted in accordance with these.
- I/we will immediately report to the HREC anything which might warrant review of the ethical approval of the proposal NS 5.5.3 including:
    - serious or unexpected adverse effects on participants;
    - proposed changes in the protocol; and
    - unforseen events that might affect continued ethical acceptability of the project.
- I/we will inform the HREC, giving reasons, if the research project is discontinued before the expected date of completion NS 5.5.6 see NS 5.5.8(b);
- I/we will adhere to the conditions of approval stipulated by the HREC and will cooperate with HREC monitoring requirements. At a minimum annual progress reports and a final report will be provided to the HREC.

Applicant / Chief Researcher(s) / Principal Researcher(s)

Prof Lucinda Chipchase<br>University of Western Sydney	___________________<br>Signature	____/____/______<br>Date
Dr Siobhan Schabrun<br>The University of Queensland	___________________<br>Signature	____/____/______<br>Date
Ms Emma Jones<br>University of Western Sydney	___________________<br>Signature	____/____/______<br>Date
Prof Vaughan Macefield<br>University of Western Sydney	___________________<br>Signature	____/____/______<br>Date

Supervisor(s) of student(s)

I/we certify that:

- I/we will provide appropriate supervision to the student to ensure that the project is undertaken in accordance with the undertakings above;
- I/we will ensure that training is provided necessary to enable the project to be undertaken skilfully and ethically.

Prof Lucinda Chipchase	___________________<br>Signature	____/____/______<br>Date
Dr Siobhan Schabrun	___________________<br>Signature	____/____/______<br>Date

Prof Vaughan Macefield ________________________________ ____/____/______
Signature Date

Heads of departments/schools/research organisation

I/we certify that:

- I/we are familiar with this project and endorse its undertaking;
- the resources required to undertake this project are available;
- the researchers have the skill and expertise to undertake this project appropriately or will undergo appropriate training as specified in this application.

_________ __________________ ________________________________
Title First name Surname

________________________________ ________________________________
Position Organisation name

____/____/____ ________________________________
Date Signature

11. ATTACHMENTS

This page and all pages that follow don't need to be submitted to your HREC.

11.1 List of Attachments

Core Attachments	Attachments which may be required/appropriate.
Recruitment/invitation	Copy of advertisement, letter of invitation etc
Participant Information	Copy or script for participant<br>Copy or script for parent, legal guardian or person responsible as appropriate
Consent Form	Copy for participant<br>For parent, legal guardian or person responsible as appropriate<br>For, optional components of the project eg. genetic sub study
Peer review	Copy of peer review report or grant submission outcome
HREC approvals	Copy of outcome of other HREC reviews

Attachments specific to project or participant group	Attachments which may be required/appropriate.
Administration of a drug for research but is not clinical research	Technical information about the drug
Clinical trial under CTN/CTX scheme	Outcome of TGA evaluation under CTX scheme<br>Compensation statement<br>Indemnity agreement<br>Insurance certificate<br>Clinical trial agreement<br>TGA forms for signature if study approved
Peer Review	If appropriate also provide copies of previous grants, reports or project proposals that are directly applicable to this ethics application.

11.2 Participant information elements
Core Elements
Provision of information to participants about the following topics should be considered for all research projects.

Core Elements	Issues to consider in participant information
About the project	Full title and / or short title of the project<br>Plain language description of the project<br>Purpose / aim of the project and research methods as appropriate<br>Demands, risks, inconveniences, discomforts of participation in the project<br>Outcomes and benefits of the project<br>Project start, finish, duration
About the investigators / organisation	Researchers conducting the project (including whether student researchers are involved)<br>Organisations which are involved / responsible<br>Organistions which have given approvals<br>Relationship between researchers and particpants and organisations
Participant description	How and why participants are chosen<br>How participants are recruited<br>How many participants are to be recruited
Participant experience	What will happen to the particant, what will they have to do, what will they experience?<br>Benefits to individual, community, and contribution to knowledge<br>Risks to individual, community<br>Consequences of participation
Participant options	Alternatives to participation<br>Whether participation may be for part of project or only for whole of project<br>Whether any of the following will be provided: counselling, post research follow-up, or post research access to services, equipment or goods
Participants rights and responsibilities	That participation is voluntary<br>That participants can withdraw, how to withdraw and what consequences may follow<br>Expectations on participants, consequences of non-compliance with the protocol<br>How to seek more information<br>How to raise a concern or make a complaint
Handling of information	How information will be accessed, collected, used, stored, and to whom data will be disclosed<br>Can participants withdraw their information, how, when<br>Confidentiality of information<br>Ownership of information<br>Subsequent use of information<br>Storage and disposal of information
Unlawful conduct	Whether researcher has any obligations to report unlawful conduct of participant
Financial issues	How the project is funded<br>Declaration of any duality of interests<br>Conspensation entitlements<br>Costs to participants<br>Payments, reimbursements to participants<br>Commercial application of results
Results	What will particpants be told, when and by whom<br>Will individual results be provided<br>What are the consequences of being told or not being told the results of

Core Elements	Issues to consider in participant information
	research<br>How will results be reported / published<br>Ownership of intellectual property and commercial benefits
Cessation	Circumstances under which the participation of an individual might cease<br>Circumstances under which the project might be terminated

Research Specific Elements
Provision of information to participants about the following topics should be considered as may be relevant to the research project.

Specific to project or participant group	Additional issues to consider in participant information
Administration of a drug for research but is not clinical research	Information about the drug, contra-indications etc
Clinical trial under CTN/CTX scheme	Explain that the trial therapy is experimental<br>Describe the phase of the trial, where applicable<br>Describe arms of trial, where applicable and explain what the trial therapy is being compared to eg. standard therapy or placebo<br>Describe the meaning of placebo, randomisation, cross-over, wash-out period etc as applicable<br>Compensation and indemnity arrangements as applicable / appropriate
